# Two-step localization driven by peptidoglycan hydrolase in interbacterial predation

**DOI:** 10.1093/ismejo/wraf208

**Published:** 2025-09-17

**Authors:** Huihui Song, Yuxiang Zhu, Zhelin Qu, Meixue Zhu, Xindong Li, Lijia Zhao, Kunpeng Wang, Ruizhen Zhang, Lei Cui, Yuying Li, Zeran Bian, Weijia Zhang, Yiliang Chen, Liangcheng Du, Jun-Lei Wang, Xian Zhao, Lu Deng, Yan Wang

**Affiliations:** MOE Key Laboratory of Evolution and Marine Biodiversity, College of Marine Life Sciences, Ocean University of China, 5 Yushan Road, Qingdao, Shandong 266003, China; MOE Key Laboratory of Evolution and Marine Biodiversity, College of Marine Life Sciences, Ocean University of China, 5 Yushan Road, Qingdao, Shandong 266003, China; Center for Optics Research and Engineering, Shandong University, 72 Binhai Road, Qingdao, Shandong 266237, China; Key Laboratory of Laser & Infrared System, Ministry of Education, 72 Binhai Road, Qingdao, Shandong 266237, China; MOE Key Laboratory of Evolution and Marine Biodiversity, College of Marine Life Sciences, Ocean University of China, 5 Yushan Road, Qingdao, Shandong 266003, China; MOE Key Laboratory of Evolution and Marine Biodiversity, College of Marine Life Sciences, Ocean University of China, 5 Yushan Road, Qingdao, Shandong 266003, China; MOE Key Laboratory of Evolution and Marine Biodiversity, College of Marine Life Sciences, Ocean University of China, 5 Yushan Road, Qingdao, Shandong 266003, China; Center for Optics Research and Engineering, Shandong University, 72 Binhai Road, Qingdao, Shandong 266237, China; Key Laboratory of Laser & Infrared System, Ministry of Education, 72 Binhai Road, Qingdao, Shandong 266237, China; MOE Key Laboratory of Evolution and Marine Biodiversity, College of Marine Life Sciences, Ocean University of China, 5 Yushan Road, Qingdao, Shandong 266003, China; MOE Key Laboratory of Evolution and Marine Biodiversity, College of Marine Life Sciences, Ocean University of China, 5 Yushan Road, Qingdao, Shandong 266003, China; MOE Key Laboratory of Evolution and Marine Biodiversity, College of Marine Life Sciences, Ocean University of China, 5 Yushan Road, Qingdao, Shandong 266003, China; MOE Key Laboratory of Evolution and Marine Biodiversity, College of Marine Life Sciences, Ocean University of China, 5 Yushan Road, Qingdao, Shandong 266003, China; MOE Key Laboratory of Evolution and Marine Biodiversity, College of Marine Life Sciences, Ocean University of China, 5 Yushan Road, Qingdao, Shandong 266003, China; MOE Key Laboratory of Evolution and Marine Biodiversity, College of Marine Life Sciences, Ocean University of China, 5 Yushan Road, Qingdao, Shandong 266003, China; Department of Chemistry, University of Nebraska-Lincoln, 1400 R Street, Lincoln, NE 68588, United States; Center for Optics Research and Engineering, Shandong University, 72 Binhai Road, Qingdao, Shandong 266237, China; Key Laboratory of Laser & Infrared System, Ministry of Education, 72 Binhai Road, Qingdao, Shandong 266237, China; Center for Optics Research and Engineering, Shandong University, 72 Binhai Road, Qingdao, Shandong 266237, China; Key Laboratory of Laser & Infrared System, Ministry of Education, 72 Binhai Road, Qingdao, Shandong 266237, China; Center for Optics Research and Engineering, Shandong University, 72 Binhai Road, Qingdao, Shandong 266237, China; MOE Key Laboratory of Evolution and Marine Biodiversity, College of Marine Life Sciences, Ocean University of China, 5 Yushan Road, Qingdao, Shandong 266003, China; Institute of Evolution & Marine Biodiversity, Ocean University of China, 5 Yushan Road, Qingdao, Shandong 266003, China; Laboratory for Marine Ecology and Environmental Science, Qingdao National Laboratory for Marine Science and Technology, 168 Wenhai Middle Road, Qingdao, Shandong 266237, China

**Keywords:** bacterial interaction, predation, localization, peptidoglycan hydrolase, signal regulation

## Abstract

Mechanisms of bacterial predation are crucial for revealing microbial adaptation strategies and interaction behaviors in the environment, yet they remain poorly understood. Previously, predators were reported to localize prey via specific cues. However, the process and mechanisms by which these cues, including signaling molecules, mediate predator localization remain unclear. Herein, we investigate the dynamic interaction between the predatory bacteria *Lysobacter enzymogenes* and its prey bacteria. By integrating genetic manipulation, transcriptomic analysis, biochemical assays, and live-cell tracking microscopy at the single-cell level, we present a novel predation strategy mediated by peptidoglycan hydrolase LssL, named peptidoglycan hydrolase-driven Prey Localization and Utilization System (phPLUS). In phPLUS, predators secrete LssL to initiate the Step I of the localization process. LssL then hydrolyzes prey and releases small molecules of glycine, which serve as signaling cues to guide the predator’s directional movement and promote the Step II of localization. In turn, prey signals upregulate the expression of LssL, which synergize with type VI secretion system to ultimately mediate prey killing through a novel regulatory pathway. This study reveals a new two-step localization strategy in bacterial predation, highlighting a previously unrecognized predation process and signal regulation mechanism, and expanding our understanding of predator–prey interactions and microbial ecological dynamics.

## Introduction

Species interactions are considered as one of the main forces sustaining the Earth’s biodiversity [1, 2] and play a pivotal role in shaping complex ecological systems [3, 4]. Of these interactions, predator–prey systems support vast and stable ecological networks [5, 6]. In microbial ecosystems, predation serves as a key strategy by which bacteria acquire nutrients [7–9], contributing significantly to population dynamics and community composition [10]. Despite its ecological importance, bacteria–bacteria predation typically involves intricate processes and signal regulatory pathways, and its intrinsic mechanisms remain poorly understood [7, 11]. Understanding how bacteria locate and kill prey is crucial for deciphering microbial interactions and ecosystem dynamics. In previously revealed predation mechanisms, specific cues from prey bacteria are sensed by predatory bacteria, which facilitates the recognition and localization of the prey, thereby achieving predation [11–13]. However, in complex natural habitats, bacterial responses to these cues, particularly signaling molecules, are influenced by various environmental factors, such as spatial distance of interspecies [14]. These variable factors increase a degree of randomness of the detection of prey cues by predators and raise an intriguing question: Do predatory bacteria possess a more efficient prey localization strategy that allows them to take the “initiative” in the complex predator–prey interactions? The processes and mechanisms underlying such advanced localization strategies remain largely unexplored.

Herein, we established a pairwise predation model using two bacterial species. *Lysobacter enzymogenes*, a Gram-negative (G^−^) predatory bacterium considered the “lyser of bacteria” [15] owing to its antagonistic effect against Gram-positive (G^+^) bacteria [16, 17], acted as the predator, and *Staphylococcus aureus*, a typical G^+^ bacterium preyed on by G^−^ predators [18] and antagonized by *L. enzymogenes* [16, 17], served as the prey. Previous studies have revealed multiple antimicrobial strategies employed by *L. enzymogenes*, including the deployment of type IV (T4SS) [19] and type VI (T6SS) [20] secretion systems to target bacteria, as well as the production of heat-stable antifungal factor (HSAF) [21] and chitinase [22] to attack fungi. These findings highlight *L. enzymogenes* as a versatile predator capable of both interspecies and cross-kingdom interaction. Notably, *L. enzymogenes* lacks flagella and exhibits motility mediated by type IV pili (T4P) [23]. This form of movement plays a critical role in prey contact and coordinated group behavior, suggesting a close link between motility and predatory function. However, it remains unclear how *L. enzymogenes* sense cues released by prey and use them to guide directional movement toward prey targets. In our study, we revealed a novel two-step prey localization strategy in bacterial predation, namely the peptidoglycan hydrolase-driven Prey Localization and Utilization System (phPLUS). In phPLUS, the peptidoglycan (PG) hydrolase (PGH) LssL and the glycine (Gly) generated through its hydrolysis of prey cell walls function as localization cues that guide predators in targeting prey, thereby mediating a two-step prey localization process. This study unveils a previously unrecognized two-step prey localization strategy in bacterial predation. It highlights the multifaceted roles of PGHs in microbial interactions and confirms the signal transduction function of the amino acid Gly in bacterial predation. These findings offer new insights into microbial interactions, particularly in the context of predation behavior.

## Materials and methods

### Bacteria strains, growth conditions, and materials

*Lysobacter enzymogenes* and mutant strains were cultivated at 28°C in 40% Trypticase Soy Broth (TSB). *Staphylococcus aureus* SYZX101 was cultivated at 37°C in Luria–Bertani (LB), *Micrococcus luteus* ATCC 4698 was cultivated at 30°C in TSB, and *Thalassospira* sp. 1–10 was cultivated at 28°C in Marine Broth 2216. *Escherichia coli* strains, including *E. coli* DH5α, *E. coli* BL21 (DE3), and *E. coli* S17–1 λpir, were employed for DNA manipulation. All molecular manipulations in this study followed previously described methods [24]. Strains and plasmids used in this study are detailed in Table S1. The necessary reagents for molecular biology and biochemistry were purchased from Sparkjade (China), Takara (Japan), and Yeasen (China). Synthesis of polymerase chain reaction (PCR) amplification primers and sequencing services were performed by Sangon Biotech (China) and Beijing Tsingke (China). A detailed list of primers is available in Table S2. Details of other assays provided in the supplementary method.

### Statistical analysis

Statistical analyses were performed by using GraphPad Prism software. The evaluation of normality is determined based on the Shapiro–Wilk test results of Normality and Lognormality Tests. Two-tailed Student’s t test, ANOVA with Tukey’s multiple comparisons test, or Binomial test was used to determine the statistical significance of differences between biological replicates in an experiment. All data showing the significance information in this study are at least three replicates. Statistical significance is represented by ns, not significant, ^*^P value < 0.05, ^**^P value < 0.01, ^***^P value < 0.001.

## Results

### Directional movement towards prey cells mediated by the peptidoglycan hydrolases LssL

**Figure 1:**
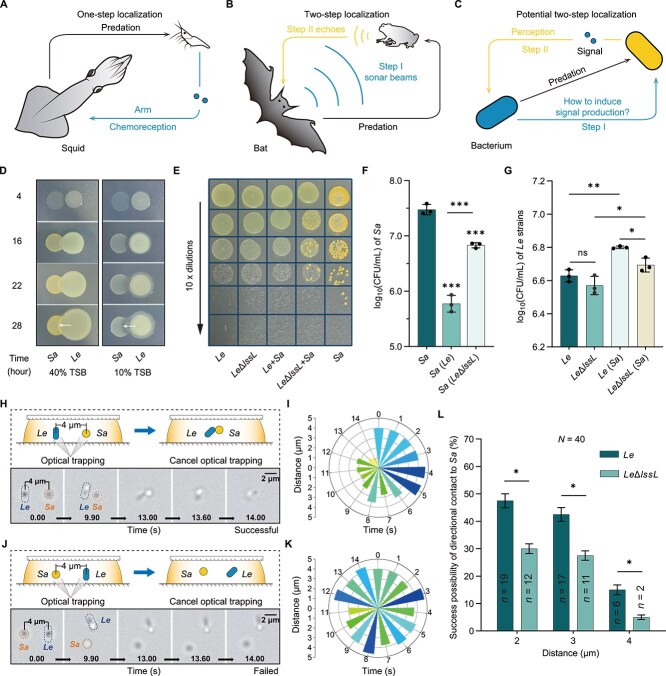
LssL involvement in the localization behavior of *L. enzymogenes* (*Le*) during its predation of *S. aureus* (*Sa*). (A) Schematic representation of the squid’s one-step localization of prey. (B) Schematic representation of the two-step prey localization process in the bat’s predation. (C) Schematic representation of the potential two-step localization of predatory bacteria toward prey bacteria. (D) The predation and invasion of *Le* community on *Sa* community. This indicates that the *Le* community is capable of migrating toward neighboring *Sa* communities. The arrow indicates the direction of movement. (E) Survival of *Le* strains and *Sa* in monocultures and pairwise co-cultures, assessed by spotting 10-fold serial dilutions of each sample on LA plates. (F) The bacterial concentration of *Sa* under monoculture and co-culture conditions, assessed by CFU assay. (G) The bacterial concentration of wild-type *Le* or *Le*Δ*lssL* under monoculture and pairwise co-culture conditions by CFU assay. (H) Schematic representation and microimaging results of the co-culture of *Le* and *Sa* at the single-cell level when they were placed at a distance of 4 *μ*M and later contacted each other. Among them, *Le* cells exhibit a rod-shaped morphology, whereas *Sa* cells are spherical. (I) Quantitative analysis of the center-to-center distance between individual *Le* and *Sa* cells in Movie S2 over a time course of 0–14 s, measured at 1 s intervals. Triangle height reflects the relative distance. (J) Schematic representation and microimaging results of failed behaviors of directional movement. (K) Quantitative analysis of the center-to-center distance between individual *Le* and *Sa* cells in Movie S3. (L) Proportion of successful movement behaviors of *Le* or *Le*Δ*lssL* toward *Sa* at the single-cell level of different original spacing. The total number of movements in each group was *N* = 40, with the number of successful behaviors denoted as *n*. for (F, G), error bars show the standard deviation (SD) of three replicates. For (L), error bars indicate the 95% confidence intervals of 40 replicates. For significance information, ns, not significant, ^*^P value < 0.05, ^**^P value < 0.01, ^***^P value < 0.001.

To unveil new predation–prey interactions, we hypothesized that the predators could adopt a more proactive and efficient strategy to locate prey cells, in contrast to passively sensing random cues released by prey cells for localization [11–13]. We sought out for inspiration from animal predation strategies. Based on previously revealed animal predations [25–27], we summarized two types of animal prey localization systems, including one-step localization (Fig. 1A) and two-step localization processes (Fig. 1B). In the one-step localization system, e.g. squids directly sense chemical signals released from prey using arm chemoreception, thereby executing a targeting ambush strategy [25] (Fig. 1A). For two-step localization, the predators not only actively locate prey cells but also induce prey to produce signaling for localization (Fig. 1B). Specifically, bats utilize a two-step localization process in their echolocation system: first, they emit sonar beams to probe their environment (Step I); subsequently, they sense the echo signals reflected by prey to precisely locate them [26, 27] (Step II) (Fig. 1B). Drawing from these examples, one-step localization can be applied to reveal bacterial prey localization mediated by specific signals [11, 12], while two-step localization offers a possible strategy for bacterial predators to more efficiently locate prey cells (Fig. 1C).

In pairwise co-cultures of *L. enzymogenes* and *S. aureus*, the prey cell concentration significantly decreased, while the predator cell concentration increased, confirming the predatory activity of *L. enzymogenes* against *S. aureus* (Fig. 1D–G). These results also demonstrated that *L. enzymogenes* exhibited directional movement toward the adjacent prey cells (Fig. 1D, Fig. S1, and Movie S1). Considering that bacterial directional movement aids in foraging and resource acquisition [28–31], the combination of sonar-like emission and directional movement of predators offers a novel perspective for exploring two-step localization in bacterial predation. We observed the directional movement dynamics between these two bacterial species at single-cell level, an extreme interacting condition, using a holographic optical tweezer (HOT) system that minimizes interference from other intraspecific cells (Fig. 1H and J). HOT technology uses laser beams to trap and move cells at the microscopic scale [32, 33]. Specifically, two bacterial cells were initially separated by different distances using HOT, and their directional movements were tracked after the optical trapping effect was canceled (Fig. 1H–L, Movies S2–S3, and Fig. S2). In the fixed microscopic field of view, bacterial movements were categorized into two types: “successful” movement behaviors, where the *L. enzymogenes* cell made physical contact with the prey cell (Fig. 1H–I and Movie S2), and “failed” behaviors, where the cells failed to make contact (Fig. 1J–K and Movie S3). We quantified the ability of *L. enzymogenes* to undergo directional movement toward the prey by examining the proportion of successful movement behaviors (Fig. 1L and Fig. S2).

Extracellular hydrolases are known as active products in bacterial interactions [34, 35]. As an important group of extracellular hydrolases, PGHs are considered effective weapons used by predatory bacteria to target the cell walls of prey bacteria [34, 36–38]. In previous studies, one of the most extensively characterized and commonly found families of PGHs is the M23 peptidase family [39, 40]. Accordingly, based on the genome of *L. enzymogenes* YC36, we searched for all proteins containing the M23 peptidase domain and identified a total of 11 candidates (Fig. S3). We characterized a novel PGH, which we named LssL (Lysostaphin-like (Lss-like) protein in *L. enzymogenes*) (Fig. S4A–C). Notably, among these candidates, only LssL possesses both a signal peptide indicating extracellular secretion, a hydrolytically active M23 catalytic domain, and an SH3b domain [41] capable of binding to the PG and drive the preferential digestion of the cell wall. This unique domain composition makes LssL a promising candidate for targeting the prey cell wall. The gene encoding LssL was cloned and the protein was purified for enzymatic activity assays, which demonstrated that LssL exhibits hydrolytic activity toward the PG of *S. aureus in vitro* (Figs S4D–G, S5 and S6A). Deletion of *lssL* weakened the capability of the extracellular secretions of *L. enzymogenes* YC36 to hydrolyze PG (Fig. S4H). These results show that LssL acts as an extracellular PGH targeting the cell wall PG of prey bacteria. Notably, the expression of *lssL* was significantly upregulated with the reduce level of nutrient within the environments (Fig. S4I), further hinting the potential role of LssL in predation. These phenomena make LssL one of the candidates for “sonar beams” and may induce the production of localization signals in the proposed two-step localization process (Fig. 1C).

Compared with the wild-type *L. enzymogenes*, the *lssL* deletion mutant significantly decreased the proportion of successful movement behaviors, based on analysis of 40 independent directional movements (Fig. 1L). This indicates that the ability of *L. enzymogenes* to approach the prey was weakened in the absence of *lssL* at the single-cell level. The above results suggest a direct involvement of LssL in the directional movement of *L. enzymogenes* toward prey bacteria. However, the absence of *lssL* did not influence the expression of genes involved in regulating T4P-mediated motility in *L. enzymogenes*, including *pilG*, *pilS*, and *pilR* (Fig. S4J), which are crucial for the positive regulation of *L. enzymogenes* motility force [42, 43]. Therefore, the role of LssL in the directional movement is not related to the motility strength of *L. enzymogenes* but rather involved in localizing prey bacteria. Taken together, LssL plays a key role in prey localization by *L. enzymogenes* and may be involved in the Step I of the two-step localization process in bacteria (Fig. 1C).

### LssL-mediated Step I in the two-step localization of *L. enzymogenes* toward *S. aureus*

**Figure 2:**
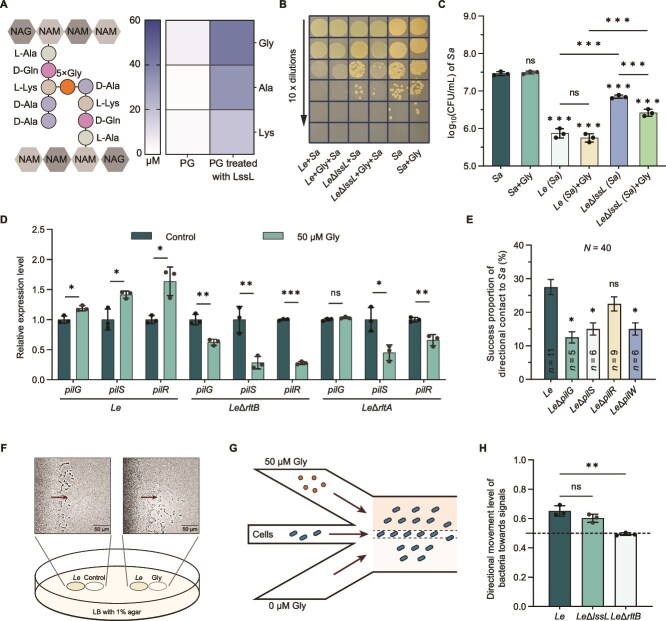
Gly induced by LssL activates the directional movement of *Le* toward *Sa*. (A) Schematic representation of the PG structure of the *Sa* cell wall, and the concentration of free Gly, Ala, and Lys of the *Sa* cell wall PG with or without LssL treatment. (B) Survival of *Le* strains and *Sa* in monocultures and pairwise co-cultures under different conditions (with or without Gly) by spotting 10-fold serial dilutions of each sample on LA plates. (C) The bacterial concentration of *Sa* when monocultured or co-cultured with *Le* strains under different conditions by CFU assay. (D) Relative expression level of *pilG*, *pilS*, *pilR* in *Le*, *Le*Δ*rltB*, and *Le*Δ*rltB* when untreated or treated with 50 *μ*M Gly. (E) The success rates of directional movement toward *Sa* were assessed for wild-type *Le* and motility-impaired mutant strains *Le*Δ*pilG*, *Le*Δ*pilS*, *Le*Δ*pilR*, and *Le*Δ*pilW*. For each group, the total movement count was *N* = 40, and the successful movements were designated as *n*. (F) Preliminary assessment of the directional movement of *L. enzymogenes* to Gly on a semi-solid agar plate. (G) Schematic diagram of a three-inlet microfluidic device. (H) Quantitative assessment of the directional movement levels of wild-type *Le*, *Le*Δ*lssL*, and *Le*Δ*rtlB* cells towards 50 *μ*M Gly. For (C, D, H), error bars indicate the SD of three replicates. For (E), error bars reflect the 95% confidence intervals with 40 replicates. For significance information, ns, not significant, ^*^P value < 0.05, ^**^P value < 0.01, ^***^P value < 0.001.

Given that LssL is involved in the Step I formation of two-step localization, we next hypothesized that the LssL-hydrolyzed products released from the prey cell walls could serve as localization signals that activate the predatory behavior and directional movement of *L. enzymogenes*. To test this hypothesis, we measured the concentrations of free amino acids that compose the PG of *S. aureus* cell wall [35, 40], including Gly, alanine (Ala), and lysine (Lys), in the hydrolyzed PG products by LssL. Among them, the concentration of Gly was the highest (Fig. 2A and Fig. S7A). Moreover, the expression of *lssL* was significantly upregulated when Gly was added at concentrations as low as 5 *μ*M (Fig. S7B). These results imply the potential signaling role of Gly.

We compared the predatory ability of the wild-type *L. enzymogenes* and *Le*Δ*lssL* on *S. aureus* when treated with or without Gly. The absence of *lssL* weakened the predatory strength, whereas the killing strength of *Le*Δ*lssL* was partially restored by the addition of Gly (Figs 1E–G and 2B–C). In the control groups, Gly addition did not affect either the survival rate of *S. aureus* or the killing strength of wild-type *L. enzymogenes* on *S. aureus* (Fig. 2B–C), and there may be a Gly concentration dependent effect. Therefore, as one of the hydrolyzed products of *S. aureus*’ PG by LssL (Fig. 2A and Fig. S7A), Gly plays a key role in the LssL-mediated bacterial predation (Fig. 2B–C), whereas its role in prey localizing is still unclear. Given the involvement of LssL in directional movement (Fig. 1L), we focused on bacterial T4P-mediated motility and its directional movement. The key genes involved in regulating T4P-motility [42, 43], including *pilG*, *pilS*, and *pilR*, were upregulated by Gly addition (Fig. 2D). At the single-cell level, mutant strains with impaired motility, including *Le*Δ*pilG*, *Le*Δ*pilS, Le*Δ*pilR*, and *Le*Δ*pilW*, exhibited reduced success rates in directional movement toward *S. aureus*, with key regulatory genes *pilG*, *pilS*, and constitutive gene *pilW* of T4P-motility showing statistically significant reductions (Fig. 2E and Fig. S8).

To investigate whether the Gly signal is involved in the directional movement of *L. enzymogenes*, we first conducted a preliminary assessment on a semi-solid agar plate (Fig. 2F). The results showed that *L. enzymogenes* exhibited directional movement toward the side containing the Gly signal (Fig. 2F), and this behavior was likely mediated by T4P-driven motility. We then employed a three-channel microfluidic device to further assess the directional movement of *L. enzymogenes* to signaling molecules (Fig. 2G). When *L. enzymogenes* was introduced into the central channel and the two side channels contained either Gly or no additive, *L. enzymogenes* exhibited significant directional movement toward the side channel containing Gly (Fig. 2G–H and Fig. S7C–E). These results show that Gly, a molecule widely present in nature, can induce the directional movement of one predator (*L. enzymogenes*) toward specific prey (*S. aureus*). Notably, the absence of *lssL* had no effect on this directional movement (Fig. 2H), which aligned with the observation that the absence of *lssL* had no impact on the expression of motility genes (Fig. S4J). However, the absence of *lssL* significantly reduced the directional movement level (Fig. 1L). Therefore, hydrolyzing prey bacteria to release Gly is the role of LssL in the directional movement of *L. enzymogenes* toward *S. aureus*, which constitutes the Step I of the two-step localization, a LssL-PG-Gly pathway.

### LssL-mediated Step II in the two-step localization of *L. enzymogenes* toward *S. aureus*

**Figure 3:**
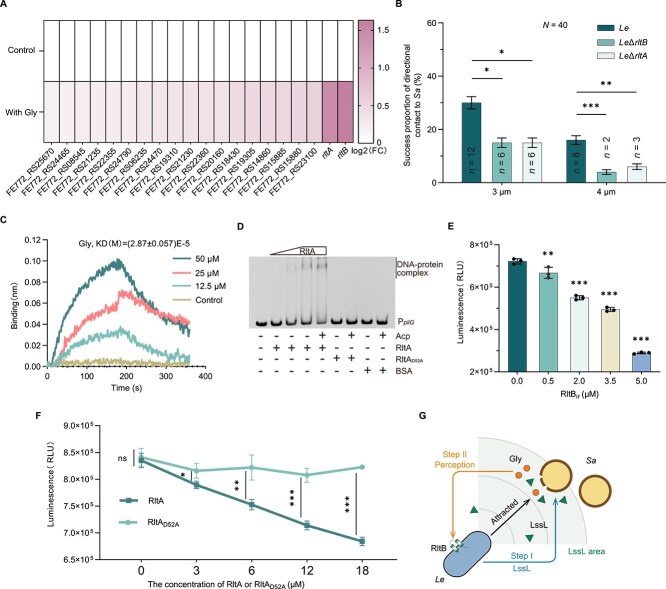
LssL-mediated Step II of the two-step localization mechanism in *Le* predation. (A) Transcriptome results of all upregulated two-component systems of *Le* by 50 *μ*M Gly. (B) Proportion of successful directional movement behaviors of *Le*, *Le*Δ*rltB*, or *Le*Δ*rltA* toward *Sa* at the single-cell level with different original spacing. Each group comprised a total of 40 movements (*N* = 40), and the successful movements were designated as *n*. (C) The binding [dissociation constant (*K*_D_)] between RltB_psrr_ and Gly. The curve of RltB_ir_ treated with 50 *μ*M Gly served as the protein control. (D) EMSA between RltA and P*_pilG_*. (E, F) Activity of RltB_ir_ kinase and phosphotransferase. The luminescence intensity is inversely proportional to kinase activity or phosphotransferase activity. For (E), the kinase activity assay of RltB_ir_. As the concentration of RltB_ir_ gradually increases, the luminescence intensity decreases, suggesting that RltB_ir_ can bind to ATP and possess histidine kinase activity. For (F), the protein phosphotransferase activity assay between RltB_ir_ and RltA or RltA_D52A_ at different concentration (0, 3, 6, 12, 18 *μ*M). As the concentration of RltA protein gradually increases, the luminescence intensity gradually decreases, indicating that RltB_ir_ can transfer phosphate groups to RltA instead of RltA_D52A_. (G) Schematic representation of the LssL-mediated two-step localization of *Le* to actively localize *Sa*. For (E, F), error bars indicate the SD of three replicates. For (B), error bars reflect the 95% confidence intervals with 40 replicates. For significance information, ns, not significant, ^*^P value < 0.05, ^**^P value < 0.01, ^***^P value < 0.001. Acp, acetyl phosphate.

We further investigated the signaling role of Gly and the mechanism of Step II. Transcriptome analysis revealed that Gly addition upregulated the expression of a series of two-component systems (TCSs) (Fig. 3A). Among these, we identified a novel TCS with the highest upregulation (Fig. 3A) and designated it as RltB/RltA (Figs S9 and S10). Both LssL-mediated PG hydrolysis products and Gly addition greatly upregulated the expression of RltB/RltA (Fig. S7F–G). The deletion of *rltB* or *rltA* abolished the Gly-induced upregulation of motility-related gene expression and even resulted in downregulation (Fig. 2D). This observation suggests that, in the absence of the primary Gly-sensing system, compensatory mechanisms may be activated, implying the existence of a more complex regulatory network underlying Gly-mediated signaling. The absence of *rltB* weakened the directional movement of *L. enzymogenes* towards Gly (Fig. 2H). Moreover, at the single-cell level, the absence of *rltB* or *rltA* significantly weakened the directional movement ability of *L. enzymogenes* (Fig. 3B and Fig. S11). These indicate that RltB/RltA plays a crucial role in Gly-induced directional motility.

We cloned and purified the relevant proteins of RtlB and RltA for biochemical detection (Fig. S6B–E). Using bio-layer interferometry and electrophoretic mobility shift assay (EMSA), we observed that the Gly bound directly to the predicted signal recognition region of RltB (RltB_psrr_), and that the P*_pilG_*, a DNA region containing the predicted promoter of *pilG*, bound directly to purified RltA (Fig. 3C–D and Fig. S12). The regulatory effect of downstream gene expression by TCSs is commonly considered to rely on the phosphorylation state of regulatory proteins [44]. Although the addition of acetyl phosphate (Acp) has been reported to phosphorylate TCS regulatory proteins *in vitro* [24, 45], the presence or absence of Acp did not affect the formation of the DNA–RltA complex, complicating the understanding of phosphorylation effects (Fig. 2D). Based on the conserved aspartic residue essential for phosphorelay in TCSs, we identified D52 of RltA as the predicted phosphorylation site (Fig. S10). RltB undergoes autophosphorylation (Fig. 3E) and subsequently transfers the phosphate group to RltA (Fig. 3F). However, the D52A mutation in RltA virtually abolished the phosphorelay between the intracellular region of RltB (RltB_ir_) and RltA (Fig. 3F). EMSA results showed that the DNA–RltA combination was abolished by the D52A mutation of RltA (Fig. 3D), indicating that this combination is dependent on the phosphorylation state of RltA. The ineffectiveness of Acp in the EMSA (Fig. 3D) likely stems from the purified RltA, retaining its original phosphorylation state owing to heterologous expression, a phenomenon also observed in other bacterial species [46]. These findings support the Gly-RltB-P-RltA-*pilG* pathway, which also confirms the localization signal role of Gly. Overall, the combination of the LssL-PG-Gly pathway (Step I) and Gly-RltB-P-RltA-*pilG* pathway (Step II) constituted the LssL-mediated two-step localization (Fig. 3G).

### T6SS predation after prey localization

**Figure 4:**
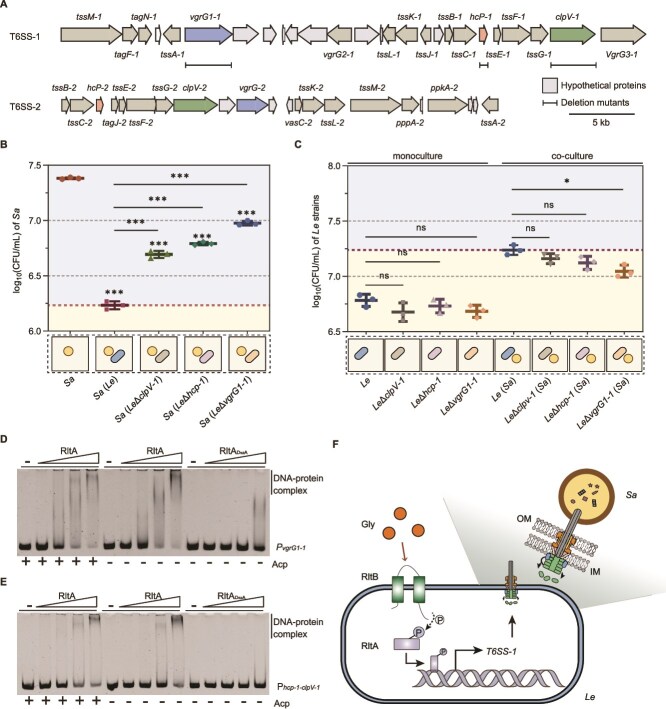
Gly promotes the killing effect of *Le* on *Sa* by enhancing the expression of T6SS-1. (A) Schematic representation of T6SS gene clusters identified in *Le*YC36. (B) Bacterial concentrations of *Sa* when monocultured or pairwise co-cultured with *Le*, *Le*Δ*clpV-1*, *Le*Δ*hcp-1*, or *Le*Δ*vgrG-1*. (C) Bacterial concentrations of *Le*, *Le*Δ*clpV-1*, *Le*Δ*hcp-1*, or *Le*Δ*vgrG-1* when monocultured or pairwise co-cultured with *Sa*. Compared with those in monocultures, the concentrations of *Le* strains in co-cultures were significantly increased. (D, E) EMSA results indicating the ability of RltA and RltA_D52A_ to bind directly to P*_vgrG1–1_* or P_*hcp-1*-*clpV-1*_. (F) Schematic representation of the killing process of *Le* through the Gly-RltB-P-RltA-T6SS-1 pathway. For (B, C), error bars indicate the SD of three replicates. For significance information, ns, not significant, ^*^P value < 0.05, ^***^P value < 0.001. OM, outer membrane; IM, inner membrane.

The evidence above supports the notion that *L. enzymogenes* activates directional movement toward *S. aureus* through two-step localization (Fig. 3G). This indicates that the predation of *S. aureus* by *L. enzymogenes* can rely on the physical contact between the two cells. T6SS is a typical contact-dependent weapon of *L. enzymogenes* in bacterial coexistence [20, 47]. We identified two T6SSs, including T6SS-1 and T6SS-2 (Fig. 4A). Notably, the expression of the key genes of T6SS-1, including *clpV-1*, *hcp-1*, and *vgrG1-1*, was significantly upregulated by the LssL-mediated PG hydrolysis products or Gly addition (Fig. S13A). However, no similar upregulation was observed for T6SS-2 (Fig. S13B). These key genes are critical for T6SS assembly and effector production [47]. The changes of *S. aureus* concentration indicated that the absence of these three genes substantially weakened the killing ability of *L. enzymogenes* against *S. aureus* (Fig. 4B). However, only the absence of *vgrG1-1* significantly decreased the concentration of *L. enzymogenes* in the pairwise co-culture with *S. aureus* (Fig. 4C). These results reflect that T6SS-1 is critical for the predatory process of prey bacteria by *L. enzymogenes*, and that the ability of *L. enzymogenes* to benefit from the bacteria killing relies on the presence of *vgrG1-1*. Thus, Gly can promote the predation of *L. enzymogenes* on *S. aureus* by upregulating the expression of T6SS-1. Further evidence from real-time PCR highlighted the essential role of the RltB/RltA pathway in this process (Fig. S13C in comparison to Fig. S13A). The deletion of *rltB* or *rltA* results in T6SS-1 related genes no longer being upregulated by Gly. EMSA results revealed that phosphorylated RltA directly binds to the DNA regions containing *vgrG1-1* promoter (P*_vgrG1–1_*) or *hcp-1-clpV-1* promoter (P_*hcp-1*-*clpV-1*_) (Fig. 4D–E and Fig. S13D). These findings align with the weakened killing ability of *L. enzymogenes* resulting from *rltB*/*rltA* absence (Fig. S13E). Therefore, following prey localization and Gly-induced attraction, the predation of *L. enzymogenes* on *S. aureus* can be mediated through the Gly-RltB-P-RltA-T6SS-1 pathway (Fig. 4F).

### Multiple roles of LssL in bacteria–bacteria predation

**Figure 5:**
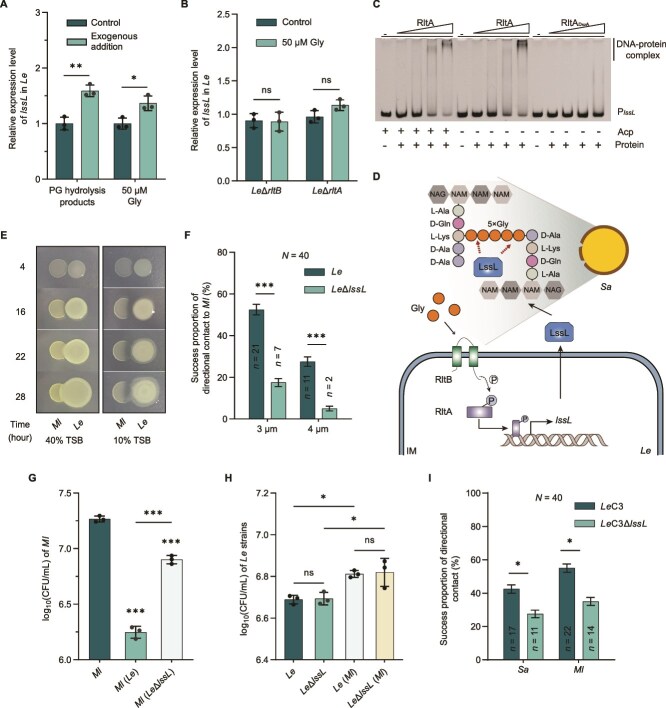
Role of LssL in the predation process of *L. enzymogenes*. (A) Relative expression level of *lssL* in *Le* under different condition (with or without PG hydrolysis products or Gly)*.* (B) Relative expression level of *lssL* in *Le*Δ*rltB* or *Le*Δ*rltA* with or without Gly. The expression of *lssL* in *Le*Δ*rltB* or *Le*Δ*rltA* was not affected by Gly. (C) EMSA between RltA or RltA_D52A_ and P*_lssL_* indicate the ability of RltA to bind directly to P*_lssL_*. (D) Schematic representation of the Gly-RltB-P-RltA-*lssL* pathway. (E) The predation and invasion of *Le* community toward *M. luteus* (*Ml*) community. (F) The proportion of successful directional movements of *Le* or *Le*Δ*lssL* toward *Ml*. (G, H), The bacterial concentrations of *Le* strains and *Ml* when monocultured and pairwise co-cultured by CFU assay. (I) Validation of LssL-mediated directional movement in *L. enzymogenes* C3. For (F, I), each group comprised a total of 40 movements (*N* = 40), with successful movements designated as *n* and error bars representing the 95% confidence intervals. For (A, B, G, H), error bars show the SD of three replicates. For significance information, ns, not significant, ^*^P value < 0.05, ^**^P value < 0.01, ^***^P value < 0.001.

As earlier demonstrated, the LssL-mediated two-step localization in *L. enzymogenes* induces the production of Gly, with Gly activating the successful directional movement and killing behaviors of *L. enzymogenes* (Figs 3G and 4F). Notably, the LssL-mediated hydrolysis products of *S. aureus* PG, or the addition of Gly, in turn promoted *lssL* expression (Fig. 5A). This result aligns with the upregulation of *lssL* detected during the co-culture of *L. enzymogenes* with *S. aureus* (Fig. S14A). These findings prompted us to investigate whether the RltB/RltA, known to sense Gly, is involved in regulating *lssL* expression. Deletion of *rltB* or *rltA* abolishes the ability of exogenously added Gly to upregulate *lssL* expression (Fig. 5B). Furthermore, EMSA assays revealed that purified RltA directly binds to the predicted *lssL* promoter region (Fig. 5C, S14B–C). Together, these results demonstrate that the feedback regulation of *lssL* can be mediated by the RltB/RltA system, thereby establishing the Gly–RltB–P–RltA–*lssL* regulatory pathway (Fig. 5D). These results raise the possibility that LssL may play multiple roles in bacterial predation and could contribute to sustaining predation through self-amplifying mechanisms, warranting further investigation.

Considering that G^−^ bacterial predator often secretes PGH to kill G^+^ prey [34], LssL can serve as a weapon in *L. enzymogenes*’ predation on *S. aureus*. This is showcased by the significant decrease of the concentration of *S. aureus* with LssL treatment (Fig. S14D), demonstrating its role as a weapon in anti-G^+^ bacteria activity. Moreover, while *S. aureus* remained unaffected in a low osmotic pressure environment (Fig. S14E), the presence of LssL drastically reduced its survival level under this condition (Fig. S14E). Thus, LssL renders the cell wall of *S. aureus* too fragile to withstand cell swelling caused by the low osmotic pressure. Although T6SS is typically regarded as a weapon against G^−^ bacteria [48, 49], it is recently reported that bacterial products with M23 peptidase domains can extend T6SS functionality to target G^+^ bacteria by breaching their PG barriers [50]. With LssL containing the M23 peptidase domain that weakens the structure of prey cell walls and further enhances the ability of *L. enzymogenes* to effectively hunt G^+^ prey, a potential cooperative between T6SS and LssL may be established.

To explore the relevance of this predatory strategy in natural environments, we investigated the interactions between *L. enzymogenes* and *M. luteus* (Fig. 5E), a common soil G^+^ bacterium whose PG contains Gly [51] and can be targeted by *Lysobacter* [52, 53]. At the single-cell level, LssL mediated the directional movement of *L. enzymogenes* toward *M. luteus* (Fig. 5F and Fig. S15), and the absence of *lssL* expression weakened the predatory ability of *L. enzymogenes* (Fig. 5G–H and Fig. S14F). Therefore, a comparable Gly-dependent mechanism may be involved in the predation of *M. luteus* by *L. enzymogenes*, enabling *L. enzymogenes* to adopt similar strategies for detecting and responding to this prey. We also extended our validation using *L. enzymogenes* C3 [42, 54], a soil isolate, and found that deletion of the *lssL* gene in this strain similarly impaired its ability to exhibit directed movement toward prey, including *S. aureus* and *M. luteus* (Fig. 5I and Fig. S16). This suggests that the predatory strategy and interaction mechanism we propose may have broader applicability across different predator–prey species. Furthermore, this LssL-mediated directional movement was also observed in the pairwise coexistence of *L. enzymogenes* and *Thalassospira* sp. (Fig. S14G and S17), a G^−^ bacterium [55, 56]. Considering the limited inhibitory effects of *L. enzymogenes* on other G^−^ bacterial species [57], the LssL-mediated two-step localization process may be involved in other types of bacterial interactions besides predations.

**Figure 6:**
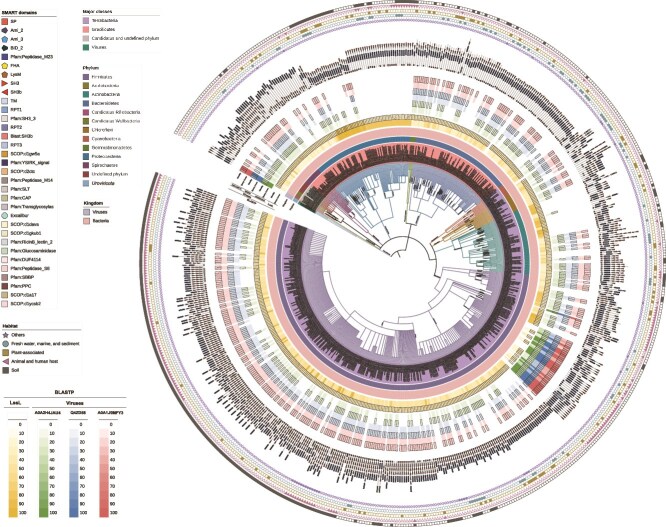
Phylogenetic tree of bacterial species and viruses that carry Lss-like proteins containing both the predicted M23 peptidase domain and SH3b domain. Bacterial species carrying Lss-like proteins are present within the phyla of major bacterial clades [59], including Gracilicutes and Terrabacteria. Clades in different colors indicate the phylum information. Round pure color blocks from the inside out show separately the classification of these microbial species at the phylum-level and kingdom-level. In the circular heat map, the sequence identity and query cover information of all other Lss-like proteins compared to LssL are shown in yellow, and the information of bacterial Lss-like proteins compared with those from viruses are shown separately in green, blue, and red. Alignments with non-significant sequence identity (*e* value > 0.05) are excluded from the heat map. The predicted domain information is showcased in the further outside part. In the outermost part, different shapes arranged in round indicate the habitat information of bacterial species, with white-filled shapes representing species with unrecorded habitats.

In addition to the predicted signal peptide region, LssL is composed of two other predicted domains: the M23 peptidase domain and the SH3b domain (Fig. S3 and Fig. 4B). These two domains constitute the mature peptidase and have been defined for Lss [39, 58], a well-studied PGH identified in *Staphylococcus simulans* [58]. Based on the UniProt database, we collected species classification information for Lss-like proteins that contain both the M23 peptidase domain and the SH3b domain (Fig. 6). The Lss-like proteins are found across multiple phyla of major bacterial subdomains (Fig. 6), including the Terrabacteria and the Gracilicutes. These two subdomains are regarded as the earliest branches diverging from the last common ancestor of bacteria [59]. Additionally, Lss-like proteins are widespread in various natural environments (Fig. 6). These findings highlight the potential ecological significance of Lss-like proteins in natural bacterial communities. We analyzed the evolutionary diversity of the M23 domain in Lss-like proteins given the conserved distribution of the domain across different Lss-like proteins (Fig. 6 and Fig. S18). Our analysis showed three main evolutionary classes (Fig. S18). Lss belongs to an independent class that may represent a more primitive lineage, compared with the other two classes (Fig. S18). However, further evolutionary analysis of Lss-like proteins suggests that these three classes may be divided, with proteins such as LssL potentially representing the most ancient class (Fig. S19). The difference between the findings from these two evolutionary analyses indicates that the evolutionary diversity of Lss-like proteins can be simultaneously influenced by both the M23 domain and the complete proteins themselves. This evolutionary complexity further supports the possible designation of multiple roles of Lss-like proteins, such as LssL, in species interactions involving bacteria.

## Discussion

**Figure 7:**
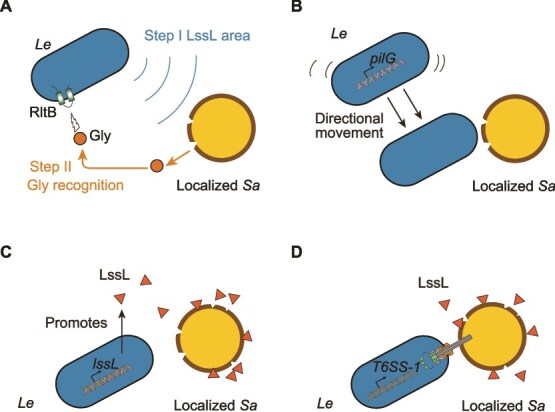
Two-step localization mechanism of *Le* toward prey. (A) Schematic representation of the two-step localization of *Sa* by *Le*. (B) Schematic representation of the directional movement of *Le* toward localized *Sa*. This process is activated by Gly. (C) The schematic representation of the enhanced expression of LssL to promote the killing effect of *Le* on *Sa*. (D) Schematic representation of the cooperation between LssL and T6SS in the killing process of *Sa* by *Le*.

In previously revealed bacteria–bacteria predations [11, 12, 34], signals specifically released by prey bacteria drive the predator–prey dynamics. It is worth noting that a previous study on social amoebae preying on bacterial cells revealed that prey bacteria can detect and counterattack predators through a system analogous to a “chemical radar” [60]. Inspired by this finding, we employed *L. enzymogenes* and *S. aureus* as a predator–prey model and discovered that predatory bacteria can also utilize an “echolocation” strategy analogous to bat radar [26, 60]. In this mechanism, predatory bacteria secrete PGH to locate prey in two steps (Fig. 2G). We termed this novel predation mechanism the bacterial “peptidoglycan hydrolase-driven Prey Localization and Utilization System” (phPLUS). The phPLUS confirms that PGH and the Gly signal produced by its hydrolysis of prey cell walls can play a localization role in bacteria-bacteria predation processes. Specifically, in bacteria–bacteria predation, the LssL-mediated two-step localization induces the production of Gly (Fig. 7A), which later activates the directional movement and killing process of *L. enzymogenes* toward *S. aureus* (Fig. 7B–C). This may in turn synergize with bacterial weapons such as T6SS-1 to optimize the predatory efficiency (Fig. 7D). Collectively, these findings underscore the integration of two-step localization and potentially cooperative predatory mechanisms, both of which depend on the versatile functions of LssL (Fig. 7A–D), and provide a novel “proactive” mechanism for bacterial predation strategies.

Because the PG of G^+^ bacterial cell walls is structurally complex, the potential roles of other compositional molecules should also be considered. Real-time PCR analysis revealed that L-Lys, L-Ala, and D-Ala significantly upregulated certain phPLUS-related genes to varying degrees (Fig. S20A), suggesting that these molecules may also contribute to the phPLUS process in *Le*YC36. Further studies are required to clarify their potential signaling roles in phPLUS and the regulatory pathways they depend on. However, Gly is widely distributed in natural environments, with concentrations ranging from tens to hundreds of nM in seawater [61] and freshwater [62], and around 10 *μ*M in soil habitats [63]. It is noteworthy that regions of intense interactions in the environment can cause significant local increases in the concentration of certain substances [64, 65]. For example, prey lysis during predation releases Gly, leading to an increase that exceeds the environmental background. Our results also indicate that Gly at levels higher than environmental concentrations has a stronger ability to upregulate LssL. Therefore, predators may thus rely on such transient and localized increases to clearly distinguish them from background concentrations. Based on real-time PCR analysis under different conditions, including the presence or absence of prey (Fig. S14A) or Gly (Fig. 5A), as well as different nutrient levels (Fig. S4I), LssL was found to be continuously expressed in wild-type *L. enzymogenes*. Without prey, LssL remained at low levels to conserve energy, whereas prey presence and local Gly increase triggered high expression, consuming energy to support predation. Therefore, this variation in expression levels helps bacteria maintain energy balance and optimize between energy expenditure and predation strategy, providing a new direction for future research. Additionally, given that this study is limited to a single species of predator, further studies on phPLUS involving other predatory bacteria and predator–prey dynamics should be expected. Previous work [7] showed that predator use “grappling hooks” to capture prey and activate T6SS, which is a short-range weapon strategy [66]. In contrast, LssL-mediated prey localization acts as a mid-range weapon [67], guiding predators to move toward prey. This highlights the diversity of bacterial predation strategies and raises questions about whether other predators also possess phPLUS, how these strategies are integrated and prioritized, and whether they act sequentially or synergistically.

This study explored prey localization by predator cells at the single-cell level; however, certain predator cells at the colony periphery may act as “scouts”. We speculated that phPLUS could be applied to the “scout” strategy of bacterial communities. The absence of *lssL* did not influence the motility (Fig. S4J) but reduced the dispersion degree of colony edge cells (Fig. S20B). We speculated that LssL could also induce the generation of unknown intraspecific signaling molecules of *L. enzymogenes* that encourage edge cells to act as “scouts” for localizing prey cells. This implies that the role of extracellular PGHs in interactions involving bacteria may extend beyond our current understanding, and that the PGH-driven two-step localization process may occur between bacteria and other non-bacterial species, or among bacteria themselves. Therefore, the mechanisms of the directional movement and social division of predator communities still require further studies. In particular, although the addition of Gly upregulated the expression of T6SS-1 (Fig. S13A), the prey-killing ability of the wild-type *L. enzymogenes* was not enhanced by Gly (Fig. 2B–C). This result may be caused by the concentration-dependent effect of signals. Specifically, the two-step localization process may generate adequate amounts of Gly, thereby rendering externally added Gly ineffective as a localization or predation cue. A similar phenomenon was also observed in the responses of *L. enzymogenes* toward spermidine [68]. Moreover, intricate intraspecies interactions and unknown upstream effects of LssL may also contribute to this result. Future studies should explore combined analyses of phPLUS, intraspecific interactions, and enzyme-driven two-step localization of different bacterial species from various environments. Furthermore, the mechanisms and evolutionary diversity of bacterial phPLUS may need to be co-analyzed with animals’ echolocation, further deepening our understanding of the predator–prey system among microbial species.

## Supplementary Material

Supplementary_material_wraf208

Dataset_S1_wraf208

Dataset_S2_wraf208

Dataset_S3_wraf208

Movie_S1_wraf208

Movie_S2_wraf208

Movie_S3_wraf208

## Data Availability

All data used in this study are available in the article and supplementary information.
