# Supplementary material for: Two-step localization driven by peptidoglycan hydrolase in interbacterial predation
Source: ISME J. 2025 Sep 17;19(1):wraf208. doi: 10.1093/ismejo/wraf208 (PMC12510463; doi:10.1093/ismejo/wraf208)
Supplement: Supplementary_material_wraf208 [file supplementary_material_wraf208.docx]

**Supplementary material**

**Two-step localization driven by peptidoglycan hydrolase in interbacterial predation**

**Supplementary** **Notes**

Naming of LssL

Based on the BLASTP results in the swissprot database and the results of SMART domain analysis, LssL (Lysostaphin-like protein in *L. enzymogenes*) is compared to Lysostaphin (P10547.2, Lss), a reported protein from *Staphylococcus simulans* that lyses staphylococcal cells by hydrolyzing the peptide bridge of peptidoglycan[41, 58, 69]. This result showed high homology (47.17% identity, 20% cover, *e* value = 2E-6). Both of LssL and Lss carry the M23 domain (catalytic domain) and the SH3b domain (PG binding domain) (Fig. S4A-B). Therefore, this extracellular peptidoglycan hydrolase gene in *Le*YC36 was named *lssL*. All BLASTP results used for gene naming in this study are listed in Dataset S3.

Naming of RltB/RltA

The BLASTP results based on swissprot database showed high homology of RltB/RltA with RstB/RstA of *E. coli* K12 (RltB, 27.79% of *E. coli* K12 RstB (P18392.2), 93% cover, *e* value = 8E-42; RltA, 50.21% of *E. coli* K12 RstA (P52108.2), 100% cover, *e* value = 1E-65). Therefore, we named it RltB/RltA (Rlt, RstBA-like two-component systems). All BLASTP results used for gene naming in this study are listed in Dataset S3.

**Supplementary figures**

**Figure S1. Movement and invasion of the *L. enzymogenes* (*Le*) toward *S. aureus* (*Sa*).** Real-time imaging was performed using a confocal laser microscope, displaying images captured every 6 hours for a total duration of 48 hours. The white arrow indicates the direction of movement of *Le*.

**Figure S2. Successful or failed behaviors of the directional movements of wild-type *L. enzymogenes* (*Le*) and *Le*Δ*lssL* toward *S. aureus* (*Sa*) at the single-cell level*.*** For the wild-type *Le*, its directional movements toward *Sa* at an initial distance of 2 μm and 3 μm were showcased in **(A)** and **(B)**. For the *Le*Δ*lssL*, its directional movements toward *Sa* at an initial distance of 2 μm, 3 μm, and 4 μm were showcased in **(C)**, **(D)**, and **(E)**.

**Figure S3. Predicted domain information of 11 proteins containing the M23 peptidase domain.** All proteins containing the M23 peptidase domain in the *L. enzymogenes* YC36 genome were screened, and a total of 11 candidate proteins were identified. Among these candidate proteins, FE772_RS22890 was named as LssL.

**Figure S4. Analysis of the domain and role of LssL. (A**, **B)**, The structural domain information of Lss (P10547.2) (**A**) and LssL (**B**) were predicted by SMART online analysis. The red rectangle represents the signal peptide domain. **(C)**, The Sec/SPI signal peptide of LssL predicted by SignalP. **(D, E)** LssL significantly reduces the turbidity of *Sa* PG. **(F, G)**, The concentration of small peptide fragments and amino acids in PG hydrolysis products was increased by LssL. **(H)**, The ability of extracellular secretions from *Le*, *Le*Δ*lssL*, and *Le*Δ*lssL*::*lssL* to hydrolyze PG. **(I)**, Relative expression level of *lssL* in wild-type *Le*. Compared with 100% TSB condition, the expression of *lssL* was significantly upregulated under reduced nutrient conditions (40% and 10% TSB). **(J)**, Relative expression level of *pilG*, *pilS*, and *pilR* in *Le* or *Le*Δ*lssL*. For **(D**, **F**, **I**, **J)**, error bars show the Standard Deviation of the Mean (SD) of three replicates. For significance information, ns, not significant, **P* value < 0.05, ***P* value < 0.01, ****P* value < 0.001.

**Figure S5. The heterologous expression strain of *lssL*.** **(A)**, The signal peptide sequence of LssL (highlighted in red). **(B**), The recombinant vector LssL-pET-28a(+) sequence constructed in *E. coli* BL21 (DE3). The start codon is highlighted with a red box.

**Figure S6. SDS-PAGE assay for purified recombinant proteins.** The recombinant vectors used for constructing protein expression strains were successfully verified by DNA sequencing. The samples of recombinant LssL (**A**), RltA (**B**), RltA_D52A_ (**C**), RltB_psrr_ (**D**), and RltB_ir_ (**E**) purified by different concentrations of imidazole were analyzed by SDS-PAGE.

**Figure S7. LssL-mediated the two-step localization of *Le* toward *Sa*. (A)**, Concentrations of free amino acids. The concentrations of free Gly, Ala, and Lys in dialysis buffer and LssL solution indicated that the free amino acids resulted from hydrolyzed PG and not from LssL. **(B)**, Relative expression level of *lssL* in wild-type *Le*. Under different concentrations of Gly addition, the expression of *lssL* was upregulated at both 5 µM and 50 µM. However, at lower concentrations of Gly, no significant effect on the expression of *lssL* was observed. **(C–E)**, Validation of flow symmetry in microfluidic device and quantification of bacterial directional movement toward signals. For **(C)**, symmetrical flow distribution in the channel region confirms proper functioning of the microfluidic device. The direction of flow is indicated by brown arrows. For (**D)**, normalized measured widths, 𝑤_𝑖_'=𝑤_𝑖_/𝑊, of the 50 μM Gly, cells, and 0 μM Gly streams, demonstrating symmetry in each observation channel, where 𝑤_𝑖_ is the width of fluid stream and *W* is the total width of the channel distribution. For **(E)**, Schematic diagram of directional movement of cells towards 50 μM Gly and 0 μM Gly. The location of the bacteria is marked with black dots. The flow direction is indicated by brown arrows. **(F, G)**, Relative expression level of *rltB* and *rltA* in wild-type *Le* following the addition of LssL-mediated PG hydrolysis products (**F**) or Gly (**G**). For **(B, D, F, G)**, error bars show the Standard Deviation of the Mean (SD) of three replicates. For significance information, ns, not significant, **P* value < 0.05, ***P* value < 0.01, ****P* value < 0.001.

**Figure S8. Successful and failed directional movements of wild-type *Le*, *Le*Δ*pilG*, *Le*Δ*pilS*, *Le*Δ*pilR*, and *Le*Δ*pilW* toward *Sa* at the single-cell level.** Each started from an initial distance of 4 μm, with (**A**) wild-type *Le*, (**B**) *Le*Δ*pilG*, (**C**) *Le*Δ*pilS*, (**D**) *Le*Δ*pilR*, and (**E**) *Le*Δ*pilW*.

**Figure S9. Predicted domain information of RltB and RltA by SMART and the predicted transmembrane regions of RltB by DeepTMHMM online analysis. (A)**, Predicted domains of RltB, including two transmembrane (TM) domains, HAMP domain, HisKA domain and HATPase-c domain. **(B)**, Predicted domains of RltA, including REC domain and Trans reg_C domain. **(C)**, Predicted transmembrane regions of RltB.

**Figure S10. Multiple sequence alignment of RltA and RstA.** The five-pointed star represents potentially conserved phosphorylation sites, including the phosphorylation site of the reported RstA (P52108) and possible phosphorylation sites of RltA and other RstA proteins. The consistency among amino acid sites were shown in purple. The deep or light shades of purple represent high or low consistency, respectively, of the amino acid sites.

**Figure S11. Successful or failed behaviors of *Le*’s, *Le*Δ*rltB*’s, and *Le*Δ*rltA*’s directional movements toward *Sa* at the single-cell level*.*** For the wild-type *Le*, its directional movements toward *Sa* at an initial distance of 3 μm and 4 μm were showcased in (**A**) and (**B**). For the *Le*Δ*rltB*, its directional movements toward *Sa* at an initial distance of 3 μm and 4 μm were showcased in (**C**) and (**D**). For the *Le*Δ*rltA*, its directional movements toward *Sa* at an initial distance of 3 μm and 4 μm were showcased in (**E**) and (**F**).

**Figure S12. Prediction of potential promoter regions.** **(A)**, The potential promoter region of *pilG*. **(B**, **C)**, Predicted promoter regions for *vgrG1-1* and *hcp-1*-*clpV-1*. **(D)**, The potential promoter region of *lssL*. The predicted potential promoter regions are marked with yellow.

**Figure S13. LssL mediated Gly-RltB-P-RltA-T6SS-1 pathway. (A)**, Relative expression level of *clpV-1*, *hcp-1*, and *vgrG1-1* in wild-type *Le* under different conditions (with or without PG hydrolysis products or Gly). **(B)**, Relative expression level of *clpV-2*, *hcp-2*, and *vgrG-2* in wild-type *Le*. **(C)**, Relative expression level of *clpV-1*, *hcp-1*, and *vgrG1-1* of *Le*Δ*rltB* or *Le*Δ*rltA* under different conditions (with or without Gly). **(D)**, The EMSA results indicate that unrelated protein BSA cannot directly bind to P*_vgrG1-1_* or P*_hcp-1_*_-_*_clpV-1_*. **(E)**, Survival of *Le* strains and *Sa* in pairwise co-cultures. For **(A–C)**, error bars show the Standard Deviation of the Mean (SD) of three replicates. For significance information, ns, not significant, **P* value < 0.05, ***P* value < 0.01, ****P* value < 0.001. Acp, acetyl phosphate.

**Figure S14. LssL-involved killing process of *Le* on *Sa* and other bacterial species. (A)**, Relative expression level of *lssL* in *Le* under monoculture and co-culture conditions. **(B)**, EMSA results between RltA or RltA_D52A_ and unrelated DNA indicate that unrelated DNA cannot bind to RltA. **(C)**, EMSA results between unrelated protein BSA and P*_lssL_* indicate that BSA cannot bind to P*_lssL_*. **(D)**, The anti-*Sa* activity of LssL. **(E)**, LssL decreased the survival level of *Sa* under the low osmotic pressure conditions (Ultrapure water). **(F)**, Survival of *Le* strains and *Ml* when monocultured and pairwise co-cultured, assessed by spotting 10-fold serial dilutions of each sample onto LA plates. **(G)**, The proportion of successful directional movements of *Le* or *Le*Δ*lssL* toward *Thalassospira* sp. (*Th*). Each group included a total of 40 movements (*N* = 40), with successful movements denoted as *n*. For **(A, D–E)**, error bars show the Standard Deviation of the Mean (SD) of three replicates. For (**G)**, error bars show the 95% confidence intervals of 40 replicates. For significance information, ns, not significant, ***P* value < 0.01, ****P* value < 0.001.

**Figure S15. Successful or failed movement behaviors of the *Le* cell or *Le*Δ*lssL* cell toward *Ml* cells.** For the wild-type *Le*, its directional movements toward *Ml* cells at an initial distance of 3 μm and 4 μm were showcased in (**A**) and (**B**). For the *Le*Δ*lssL*, its directional movements toward *Ml* cells at an initial distance of 3 μm and 4 μm were showcased in (**C**) and (**D**).

**Figure S16. Successful or failed movement behaviors of the wild-type *Le*C3 cell or *Le*C3Δ*lssL* cell toward *Sa* or *Ml* cells at a 4 μm initial distance.** (**A**) and (**B**) showed the directional movements of *Le*C3 and *Le*C3Δ*lssL* toward *Sa*. (**C**) and (**D**) showed the directional movements of *Le*C3 and *Le*C3Δ*lssL* toward *Ml*.

**Figure S17. Successful or failed behaviors of *Le*’s and *Le*Δ*lssL*’s directional movements toward *Th* at the single-cell level.** (**A**) and (**B**) showed the directional movements of wild-type *Le* toward *Th* at the initial distance of 4 μm and 5 μm. (**C**) and (**D**) showed the directional movements of *Le*Δ*lssL* toward *Th* at the initial distance of 4 μm and 5 μm.

**Figure S18. Phylogenetic tree of the M23 domain of Lss-like proteins.** Based on the M23 domain sequences of Lss-like proteins, this phylogenetic tree was constructed through the maximum likelihood method with 1000 bootstrap iterations. The innermost circular color blocks are shown in three classes in this tree, including purple, yellow, and green, which separately represent three major types. Sequentially from the inside out, the domains of the complete protein, the major classes for species classification, and the phylum information of species carrying the Lss-like protein are presented respectively. Colors of clades represent different phylum information.

**Figure S19. Phylogenetic tree of Lss-like proteins.** This phylogenetic tree of the complete Lss-like protein sequences was constructed using the maximum likelihood method with 1000 bootstrap iterations. The innermost circular color blocks represent the three classes as shown in Fig. S18. Sequentially from the inside out, the domains of complete protein, the major classes for species classification, and the phylum information of species are displayed respectively. The clades of the different phyla are shown in different colors.

**Figure S20. Regulatory effects of other amino acids from PG on the expression of predation-related genes and the role of *lssL* in colony edge cell dispersion.** (**A**) Effects of L-Lys, L-Ala, and D-Ala (each at a final concentration of 50 µM) on the expression of predation-related downstream genes in *Le.* **(B)** Compared with that of the wild-type *Le*, the dispersion degree was decreased in *Le*Δ*lssL* but was restored in *Le*Δ*lssL*::*lssL*. The white arrow indicates the direction of movement. For (**A)**, error bars show the Standard Deviation of the Mean (SD) of three replicates. For significance information, ns, not significant, **P* value < 0.05, ***P* value < 0.01, ****P* value < 0.001.

**Supplementary Methods**

**Generation of gene deletion and complementation**

Gene deletion and complementation mutant strains of *L. enzymogenes* were constructed as previously described[24, 70]. Using the *Le*YC36 genome (GCF_005954665.1) as a template, the upstream and downstream homologous arm fragments of the target gene were obtained through PCR. These fragments were connected to the pEX18Gm plasmid using the double enzyme digestion or seamless cloning, and the recombinant plasmid was sequentially transformed into *E. coli* DH5α and *E. coli* S17-1 λpir receptor cells using the heat shock method. According to the previously described protocol[24], *E. coli* S17-1 λpir cells harboring the recombinant vector containing homologous arm were used as the donor strain for conjugation with *Le*YC36. After the genome recombination, the gene deletion mutant strains were verified through PCR. The construction of gene complementation requires the successful construction of gene deletion mutants. The recombinant plasmid carrying the upstream-gene-downstream sequence was used for the construction of complementation strains. The transformation, recombination, and verification methods are the same as above.

**Recombinant protein expression and purification**

The DNA sequences of LssL, RltA, RltB_psrr_, and RltB_ir_ were amplified from *Le*YC36’s genomic DNA. The products were ligated respectively into the pET-28a(+). For LssL, the signal peptide sequence was deleted. Next, the recombinant plasmids were transformed into *E. coli* BL21 (DE3) for protein expression. These expression strains were grown in LB (100 μg/mL kanamycin) at 37℃ to OD_600_ = 0.6. Then, isopropyl β-D-thiogalactoside (IPTG, final concentration of 0.1 mM) was added into the bacterial suspensions, and they were grown at 16℃ for 16 hours. After the centrifugation (8000 rpm, 10 minutes), cells were collected and later resuspended in the binding buffer (20 mM Tris-HCl, 0.5 M NaCl, pH 8.0). By using an ultrasonic cell pulverizer (JY92-IIN, Scientz, China), the cells were crushed thrice (35% power, 10 minutes) and later centrifuged at 4℃ (12000 rpm, 10 minutes). The supernatant was used to purify recombinant proteins, using Ni-NTA Agarose (QIAGEN, Germany) and eluted buffers (20 mM Tris-HCl, 0.5 M NaCl, 10/20/50/75/100/250/500 mM imidazole, pH 8.0). To remove imidazole from protein samples, LssL was treated with dialysis buffer (20 mM Tris-HCl, pH 8.0, 0.85% NaCl), RltA, RltA_D52A_, RltB_psrr_, and RltB_ir_ were treated with dialysis buffer (20 mM Tris-HCl, pH 7.0, 100 mM KCl, 50 mM NaCl, 2 mM EDTA, 10% glycerol, 0.5% Tween 20). The protein purity degree was determined using sodium dodecyl sulfate-polyacrylamide gel electrophoresis (SDS-PAGE) (Fig. S6), and the concentration was determined through Bradford Protein Assay Kit (Beyotime, China).

For site-directed mutation, RltA_D52A_-pET-28a(+), the recombinant plasmid, was amplified from RltA-pET-28a(+) with site-directed mutation primers (Table S2) and repaired with *E. coli* DH5α. Next, this mutant plasmid was transferred into *E. coli* BL21 (DE3), and the mutant proteins was purified using the earlier described method.

**Peptidoglycan extraction**

Using previous methods with some modifications[71–73], peptidoglycan (PG) was extracted from *S. aureus* cells. Specifically, *S. aureus* was grown to exponential phase in LB and later resuspended at 1:10 (W: V) in the buffer (10 mM HEPES, pH 7.0, 0.3% SDS). This mixture was treated at 80℃ for 2 hours to remove teichoic acids. Following centrifugation (14000 rpm, 10 minutes), the precipitate was collected and was resuspended at 1:20 (W: V) with the wash buffer (0.02 M sodium acetate with pH 4.6, chloroform, and methanol at a volume ratio of 4:5:10) to remove lipids. The precipitation products from the centrifugation (14000 rpm, 10 minutes) were collected and later treated at 1:20 (W: V) with 0.1 M Tris-HCl buffer (pH 7.5) containing 1 mg/mL trypsin to remove proteins. Finally, PG precipitate was suspended in ultrapure water and after vacuum freeze-drying (FD-1A-80, Boyikang, China), insoluble PG powder of *S. aureus* was obtained.

**Turbidity reduction assay**

Turbidity reduction assay was based on the previous methods[74] with some modifications. After overnight culture, the OD_600_ of *Le*YC36, *Le*Δ*lssL*, and *Le*Δ*lssL*::*lssL* was adjusted to 1.0. The samples were transferred to 40% TSB at a rate of 1% and incubated at 28℃ for 24 hours. After the centrifugation (10000 rpm, 10 minutes) to remove bacterial cells, the extracellular secretions were obtained using a sterile filter (13 mm/0.22 μm, Navigator, China). The PG of *S. aureus* at a final concentration of 3 mg/mL was added to the extracellular secretions, and the decrease degree of PG turbidity at 28℃ was detected using Microplate Reader (Cytation 5, BioTek, USA). The OD_600_ value was measured every 5 minutes for 2 hours. All these experiments were performed with three biological replicates.

**LssL’s antibacterial activity assay**

*S. aureus* was grown to exponential phase in LB. After centrifuging at 6000 rpm for 5 minutes, the supernatant was removed. The control group was treated with dialysis buffer, while the experimental group was treated with 8 μM LssL. After culturing at 28℃ for 24 hours, the antibacterial activity was calculated by measuring the OD_600_.

For bactericidal experiments under low osmotic pressure conditions, the control group was treated with dialysis buffer, while the experimental group was treated with 8 μM LssL. After culturing at 28℃ for 5 hours, the OD_600_ of *S. aureus* was adjusted to 0.35, followed by centrifugation. The experimental group was treated with sterile ultrapure water, while the control group was treated with physiological saline (0.85% NaCl). These mixtures were incubated at 37℃ for 24 hours, and the relative survival rate of the bacteria was calculated by measuring the OD_600_.

**Hydrolysis product assay**

The previously described method was applied in this study with some modifications[34]. *S. aureus* PG (final concentration of 2 mg/mL) was added into the dialysis buffer and LssL solution (final concentration of 8 μM), respectively. Dialysis buffer and LssL solution (without adding *S. aureus* PG) were used as negative controls. These mixtures were incubated at 28°C for 24 hours. Then, the LssL protein and unhydrolyzed PG were removed through ultrafiltration and centrifugation, and the PG hydrolysis products were obtained. The concentration of free amino acids in the sample was measured with the automatic amino acid analyzer (L-8900, Hitachi, Japan). In this process, the samples were dried with a Termovap Sample Concentrator (NDK200-1N, MIULAB, China) and later redissolved in 0.02 N HCl. Finally, the samples were filtered (13 mm/0.22 μm, Navigator, China) and then tested.

**Dispersion degree assay of colony edge cells**

This method is based on the previously described methods[42, 75] with some modifications. *Le*YC36, *Le*Δ*lssL*, and *Le*Δ*lssL*::*lssL* were grown in 40% TSB overnight at 28℃. The OD_600_ value of these bacterial suspensions was uniformly adjusted to 1.0. Then, they were inoculated at the edge of the sterilized coverslip containing Luria Agar (LA) with 0.8% agar and were later grown at 28℃ for 24 hours. Using the inverted microscope (×400) (XD-101, JIANGNAN, China), the dispersion degree of bacterial colony edge cells was obtained. The image color was then processed to grayscale using WPS Office software.

**Predation and invasion between bacterial communities**

This was based on the previous distance predation assays[10, 76], with some modifications. After overnight culture, bacterial predator and prey were adjusted to the same value (OD_600_ = 1.0). Next, 5 μL of prey and predator suspensions were separately placed on the 10% or 40% Tryptose Soya Agar (TSA) containing 1% agar. They were grown at 28℃, and the colony status of these two communities at different times was observed and recorded. In addition, a confocal laser microscope (SDC-G, ECHO, USA) was used to conduct 48-hour time-lapse imaging of *Le*YC36 and *S. aureus* in the region, with images captured every 2 hours compiled into Movie 1 and those captured every 6 hours compiled into Fig. S1.

**Bacterial predation assay**

The bacterial predation assay in this study was based on the previously described method with some modifications[77]. Predators (*Le*YC36, *Le*Δ*lssL*, *Le*Δ*rtlB*, *Le*Δ*rtlA*, *Le*Δ*rtlBA*, *Le*Δ*clpV-1*, *Le*Δ*hcp-1*, and *Le*Δ*vgrG1-1*) were sub-cultured to OD_600_ = 1.0 in 40% TSB. Prey bacteria (*S. aureus* and *M. luteus*) were cultured to OD_600_ = 1.0 in their respective suitable media. Bacterial suspensions of *S. aureus* and *M. luteus* were concentrated 5-fold. Then, each predator suspension and prey suspension were co-cultured pairwise in physiological saline (0.85% NaCl) at a ratio of 1% at 28℃ for 24 hours. Each sample was subjected to 10-fold serial dilutions with physiological saline (0.85% NaCl). Then, the 5 μL of each diluted sample was cultured on solid LA media in rows. For colony-forming units (CFU) assay, the 120 μL of each diluted sample was plated on solid LB plates with 50 μg/mL kanamycin to measure predator’s concentrations and plated on the solid LB plates without kanamycin to measure the prey’s concentrations. After being grown at 28℃ for 48 hours, the number of bacterial colonies was counted, and the log_10_(CFU/mL) results were measured.

**Optical tweezers detection of interaction behavior**

A holographic optical tweezer (HOT) system was employed to microscopically observe the proposed mechanism by manipulating and detecting interaction behavior[33, 78].

**1. HOT configuration.** The system begins with a continuous-wave solid-state laser, operating at a wavelength of 1064 nm and an M^2^ value of less than 1.2 (MFSC-30W-CEA1.0, Maxphotonics, China). The laser beam is polarized by a linear polarizer (GL10-C26, Thorlabs, USA) and subsequently expanded to form a beam waist radius of 4 mm, corresponding to the input aperture of the spatial light modulator (SLM) (PLUTO-2.1-NIR-149, HOLOEYE, Germany). The surface of the SLM is conjugated to the back focal plane of an objective lens with a numerical aperture of 1.2 (CFI Plan Apochromat VC 60xA WI, Nikon, Japan), mounted on an inverted microscope (Ti-2, Nikon, Japan). The microscope allows multiple observation techniques, including diascopy, fluorescence, confocal laser scanning microscopy, etc. This configuration allows the creation of an optical trap with a highly focused Gaussian laser beam. Moreover, the SLM enables the generation of multiple optical traps through wavefront modulation. Specifically, to create an optical trap at position (*x*, *y*, *z*) near the objective’s focal point, the phase loaded on the *j*-th pixel of the SLM, $\phi_{j}$, is

$$\phi_{j}=\frac{\pi z}{\lambda f^{2}}\left( x_{j}^{2}+y_{j}^{2} \right)+\frac{2\pi}{\lambda f}\left( x_{j}x+y_{j}y \right)$$

where *λ* is the laser wavelength, *f* is the focal length of the objective, and (x*_j_*, y*_j_*) are the coordinates of the *j*-th pixel on the SLM. When multiple optical traps are required, phase values of each focal point are superimposed and optimized using iterative algorithms such as the weighted Gerchberg-Saxton algorithm. The algorithm, while effective, introduces significant computational complexity. To address this, parallel calculations utilizing a graphics processing unit (GPU) were employed to ensure rapid computation and responsive manipulation of the multiple optical traps.

**2. Sample preparation.** The sample for microscopic observation of interaction consists of a mixture of predator (*Le*YC36, *Le*Δ*lssL*, *Le*Δ*rtlB*, *Le*Δ*rtlA*, *Le*Δ*pilG*, *Le*Δ*pilS*, *Le*Δ*pilR*, *Le*Δ*pilW*, *Le*C3, and *Le*C3Δ*lssL*) and prey (*S. aureus*, *M. luteus*, and *Thalassospira* sp.) in 10% TSB. Each strain was adjusted to an OD_600_ of 0.6 and subsequently 10 μL of both predator and prey were added to 1 mL of 10% TSB to achieve the final working cell density. The sample fluid, with a nominal volume of 50 μL, is housed in micro-chambers formed from slide glasses and cover glasses, which are sealed together using double-sided adhesive tape.

**3. Detection and observation of interaction.** To observe the interaction between individual bacteria using the HOT system, a predator and a prey were brought within a specific distance of micrometers and then released. The behavior of the predator was tracked using the microscope, with predators and prey distinguished based on their differences in cell morphology. For each group, the experiment was repeated 40 times with different bacteria in the sample chamber. Using the Binomial test for data processing, a significance analysis was performed on the number of successful directional movements in 40 groups.

**4. Bacterial spacing analysis.** Frames were extracted every 1 second from the 0th to the 14th second of Movies S2–S3 using the OpenCV package. Positions of two bacterial centers in each frame were manually localized using ImageJ, thus obtaining the coordinate information. Then, the relative distance between the predator and prey were obtained by analyzing coordinate data.

**5. Image processing.** Screenshots of each movie at specific time points were treated with Microsoft Office PowerPoint. In this process, the brightness of screenshots was adjusted to 50%-70%, and the color temperature was adjusted to 6000. The movies in the Fig.1 involving the displayed duration are present in Movies S2–S3.

**Bacterial** **directional migration**

For directional movement analysis on semi-solid agar plates, *Le*YC36 was cultured overnight and adjusted to an OD_600_ of 1.0. Then, 2 μL of bacterial suspension and a solution with or without Gly were placed separately on LA medium containing 1% agar, and cell movement near the contact edge was observed after 24 hours.

Furthermore, we modified previous methods[79, 80] to design and fabricate a chip with three-channel for directional movement of bacterial and migration experiments. The width of the middle channel at the inlet was 0.2 mm with a length of 8 mm, while the side channels were 0.4 mm wide and 6 mm long. The width from the convergence point of the three inlets to the end of the channel was 0.9 mm, with a length of 25 mm. The total channel height was 50 μm. The three-channel microfluidic mold was fabricated using standard soft lithography techniques. Microfluidic channels were cast with polydimethylsiloxane (PDMS) prepolymer and curing agent at a 10:1 ratio, poured into the master mold, and cured at 65℃. After curing, the PDMS sheet was cut and peeled off, followed by hole punching and bonding to a glass slide using oxygen plasma treatment.

The bacterial directional migration experiment was modified based on the previous methods[80, 81]. The three inlets of the microfluidic channel were respectively supplied with 10% TSB containing or lacking 50 μM Gly (side channels) and cells suspended in 10% TSB (OD_600_ = 1.0, middle channel). Syringe pump (LSP01-3A, LONGER, China) created a laminar flow of the three solutions, with the middle channel flow rate set at 0.1 μL/min and the side channels at 0.5 μL/min, maintaining a flow width ratio of 5:1:5. Imaging was performed using an inverted fluorescence microscope (10 × or 20 × objective) (IX73, OLYMPUS, Japan). For initial tests, the *Le*YC36 strain expressing mCherry was used, and channel widths were normalized for statistical analysis. For formal experiments, wild-type *Le*YC36, *Le*Δ*lssL*, and *Le*Δ*rtlB* strains were used. Three solutions were subjected to flow stratification for at least 15 minutes. After stopping the flow, the directional movement and migration of cells were observed within 30 minutes. Each strain underwent three independent replicates, with three regions sampled during each replicate. The average directional movement level from the three regions was used for statistical analysis, and the significance was assessed. The directional movement level of bacteria towards signals was calculated as follows:

$$Directional movement level of bacteria towards signals$$

$$=\frac{Number of cells migrating toward the signal}{Number of cells migrating (toward the signal + toward no signal)}$$

**Gene transcription detection**

Under experimental treatments (4% PG hydrolysis products or 50 μM Gly) or control treatment (equivalent volumes of dialysate or ultrapure water), *Le*YC36, *Le*Δ*lssL*, *Le*Δ*rtlB*, and *Le*Δ*rtlA* were grown in 40% TSB. Then the RNA was extracted through MolPure Bacterial RNA Kit (Yeasen, China). For the gene transcription assay of co-culture samples, the wild type *Le*YC36 and *S. aureus* were grown and later adjusted to the same value (OD_600_ = 1.0). Afte being concentrated 5-fold, 50 μL of *S. aureus*’ suspension was transferred onto the LA plate. Then, 50 μL of *Le*YC36’s suspension was also added to cover *S. aureus*’ community, and they were separated through microporous filter membrane (Aquo-system 50 mm/0.22 μm, SHANGHAI XIN YA, China). After being grown at 28℃ for 24 hours, RNA of these samples was extracted through MolPure Bacterial RNA Kit (Yeasen, China). For gene transcription assays under different nutritional conditions, *Le*YC36 was cultured under 10% TSB and 40% TSB, while the control group was cultured under 100% TSB. RNA was then extracted for analysis. For the regulatory effects of other amino acid molecules (L-Lys, L-Ala, and D-Ala) in PG, *Le*YC36 was grown in 40% TSB with or without a final concentration of 50 μM amino acids, and then RNA was extracted.

Then, genomic DNA was removed from RNA samples and total cDNA was synthesized by All-In-One 5 × RT MasterMix (abm, Canada). Real-time PCR was performed in a total reaction volume of 20 μL with Hieff qPCR SYBR Green Master Mix (Yeasen, China) and the StepOne Real-time PCR System (Applied Biosystems, USA), and 16S rRNA gene was used as the reference gene. All experiments were collected from three biological replicates.

**Transcriptional analysis**

Analysis of the transcriptome of *Le*YC36 under 50 μM Gly-treated and untreated conditions was performed at Scientsgene Company, Qingdao, China. Bioanalyzer 2100 (Agilent, USA) and NanoDrop 2000 (Thermo Scientific, USA) were used to determine and quantify RNA quality. After library construction and quality testing, sequencing was performed using the Illumina HiSeq 2500 platform and paired-end reads were generated. High quality clean data were obtained by quality control of raw data. All the downstream analyses were based on clean data with high quality. Then, the mapped data were obtained by sequence alignment with the *Le*YC36 reference genome. Tophat2 tools software were used to map with reference genome. Quantification of gene expression levels were estimated by Fragments Per Kilobase of transcript per Million mapped fragments (FPKM). Differential expression analysis of two conditions was performed using the DESeq R package (1.10.1). The resulting *P* values were adjusted using the Benjamini-Hochberg approach for controlling the false discovery rate. The significantly upregulated genes were defined according to the standard (log2(Fold Change) > 0, *P* value < 0.05). Transcriptome analysis results of Gly treatment used in this study are listed in Dataset S1.

**Bio-layer interferometry (BLI)**

This method was based on the previous methods with some modifications[82, 83]. The bio-layer interferometry assay was performed with the Octet R8 System (Sartorius, Germany) and PBS (0.02% Tween-20) acting as the running buffer at 25℃. The Ni-NTA biosensors (Sartorius, Germany) were equilibrated in running buffer and later loaded with the running buffer containing RltB_psrr_. The baseline, association (without and with Gly), and dissociation were sequentially performed toward biosensors loaded with or without RltB_psrr_. Using RltB_ir_ as a control, the same experimental steps as RltB_psrr_ were conducted. The data and binding parameters were analyzed using the Octet Analysis Studio software.

***In vitro* autophosphorylation and phosphotransfer assays**

This method followed the methods previously described with some modifications[84]. The Kinase-Lumi Plus Luminescent Kinase Assay Kit (Beyotime, China) was used to detect the phosphate group transfer process of RltB/RltA. The principle is based on the ATP-dependent luciferase-catalyzed luminescence reaction. In this process, the less ATP remaining, the lower the luminescence intensity. The 5 × phosphate kinase buffer (50 mM Tris-HCl, 100 mM KCl, 5 mM MgCl_2_, 10% glycerol, pH 8.0) was prepared. Each black 96-well plate was added with 10 µL of 5 × phosphokinase buffer, 2 µL of 0.5 mM ATP, and different concentrations of RltB_ir_. The total reaction system is 50 µL. Then, each sample well was added with 50 µL of detection reagent. After the reaction at room temperature for 10 minutes, chemiluminescence detection was performed using Microplate Reader (Cytation 5, BioTek, USA).

According to the reported phosphorelay between of the two component proteins of two-component systems[44], if the histidine residue of the RltB_ir_ is autophosphorylated, the phosphate group will be transferred to the aspartate residue of the RltA. Then, the concentration of ATP in the reaction system containing the gradient concentration of RltA or RltA_D52A_ was measured to determine whether the phosphate group was delivered normally. Each black 96 well plate was added with 10 µL of 5 × phosphokinase buffer, 2 µL of 0.5 mM ATP, 5 µM of RltB_ir_ and different concentrations of RltA or RltA_D52A_, and the total phosphate transfer reaction system is 50 µL. Then, each well was added with 50 µL of detection reagent.

**Electrophoretic mobility shift assay (EMSA)**

Based on the previously described method[85], some steps were modified. The purification of RltA and RltA_D52A_ were performed as described above.

The promoter sequences were predicted through the promoter prediction website (http://lin-group.cn/server/iPro54-PseKNC[86], https://www.fruitfly.org/index.html[87]). Promoter regions used in this study were showcased in Fig. S12. About 200 bp DNA fragments (target DNA and unrelated DNA) in the promoter region were amplified from *Le*YC36’s genome by related primers. They were purified with Gel/PCR Purification Kit (Sparkjade, China) and diluted to 50 ng/μL.

The 20 μL of EMSA reaction system included RltA or RltA_D52A_ proteins (0, 0.5, 1, 1.5, or 2 µg), 4 μL of 5 × EMSA/Gel Shift binding buffer, 1 μL of DNA fragments, and ultrapure water. If necessary, 1 μL of 600 mM acetyl phosphate was added in this system. After being incubated at 25℃ for 30 minutes, the free DNA and the DNA-protein complexes of these samples were separated through the 6.5% non-denaturing polyacrylamide gel at the constant voltage of 110 V for 90 minutes. Then, the gel was treated with the solution containing YeaRed Nucleic Acid dye solution (Yeasen, China) for staining, and the results were observed with the gel imaging system (JY04S-3E, JUNYI, China).

**Bioinformatics**

**1. Gene identification and conserved sites analysis.** Based on databases of the National Center for Biotechnology Information (NCBI), LssL, RltB, and RltA were identified via BLASTP. The BLASTP results used for gene naming in this study are listed in Dataset S3. Based on the UniProt database, homologous proteins annotated as RstA (compared to RltA, identity > 50%, *e* value < 1E-50) were used to multiple sequence alignment (https://www.uniprot.org/align). The online tool for Simple Modular Architecture Research Tool (SMART) (http://smart.embl-heidelberg.de/)[88] and DeepTMHMM (https://dtu.biolib.com/DeepTMHMM)[89] were used to predict protein domains. Signal peptide was detected by the SignalP program (https://services.healthtech.dtu.dk/services/SignalP-6.0/)[90].

**2. Phylogenetic tree.** Based on the UniProt database and all stable Ensembl proteomes, proteins containing simultaneously both the Peptidase_M23 and SH3b domains predicted by SMART[88] were searched for. These proteins are Lss-like proteins. SMART used the procedure of clustering proteins by species to reduce the high redundancy present in its database. The longest member of each cluster is used as a “representative” and is also the source of the proteins included in this research domain query[88]. For these Lss-like proteins, unconventional proteins (a total of 10 proteins) with the sequence length > 750 AA were removed from them, and the selected data (a total of 616 proteins) were used for subsequent analyses.

Based on the NCBI taxonomy of Lss-like proteins’ affiliated species[88, 91], the phylogenetic tree of these species was generated. For the heat map of this tree, the identity information of Lss-like proteins compared to LssL was obtained through BLASTP. The same method was also used for Lss-like proteins of viruses. The domain information predicted by SMART was collected and showcased in the tree. According to the database of the Integrated Microbial Genomes & Microbiomes database (IMG/M, https://img.jgi.doe.gov/m/)[92], the habitat information of bacterial species carrying Lss-proteins was also searched for and showed in the tree. The resource data of this tree are listed in Dataset S1.

For the phylogenetic tree reflecting the evolutionary distance of Lss-like proteins, mafft v7.505[93] was used for sequence alignment (option “--maxiterate 1000 --globalpair”). Then, Trimal v1.4[94] was used to remove non-alignment regions from the sequence alignment (option “-automated1”). Phylogenetic trees were finally constructed by using the maximum likelihood tree building tool IQ-TREE v2.1.4[95] (option “-m MFP -B 1000 --bnni -T AUTO”). Considering that all Lss-like proteins contain only one M23 domain (based on SMART prediction results), a phylogenetic tree of the M23 region of Lss-proteins was constructed using the same procedure. The visualization of all phylogenetic trees in this study was performed using iTOL (https://itol.embl.de/)[96]. The resource data used to construct the phylogenetic tree of the system are listed in Dataset S2.

**Supplementary Tables**

Table S1. All bacterial strains and plasmids used in this study. Including laboratory storage and obtained in this study.

| **Strains** | **Relevant characteristics** | **Source** |
| --- | --- | --- |
| *L. enzymogenes* YC36 | Wild-type strain | Lab storage |
| *L. enzymogenes* C3 | Wild-type strain | Lab storage |
| *S. aureus* SYZX101 | Wild-type strain | Lab storage |
| *Le*YC36-mCherry | *Le*YC36 carrying pBBR1MCS-5-Pgen-mCherry plasmid | Lab storage |
| *M. luteus* ATCC 4698 | Wild-type strain | Lab storage |
| *Thalassospira* sp. 1-10 | Wild-type strain | Lab storage |
| *E. coli* DH5α | Competent cells for DNA manipulation | Lab storage |
| *E. coli* BL21 (DE3) | *E. coli* strain for protein expression | Lab storage |
| *E. coli* S17-1 λpir | *E. coli* strain used as bacterial conjugal donor | Lab storage |
| *Le*Δ*lssL* | *lssL-*deletion *Le*YC36 mutant strain | This study |
| *Le*C3Δ*lssL* | *lssL-*deletion *Le*C3 mutant strain | This study |
| *Le*Δ*lssL*::*LelssL* | *lssL-*complementation *Le*YC36 mutant strain | This study |
| *Le*Δ*rltB* | *rltB-*deletion *Le*YC36 mutant strain | This study |
| *Le*Δ*rltA* | *rltA-*deletion *Le*YC36 mutant strain | This study |
| *Le*Δ*rltBA* | *rltBA-*deletion *Le*YC36 mutant strain | This study |
| *Le*Δ*clpV-1* | *clpV-1-*deletion *Le*YC36 mutant strain | This study |
| *Le*Δ*hcp-1* | *hcp-1-*deletion *Le*YC36 mutant strain | This study |
| *Le*Δ*vgrG1-1* | *vgrG1-1-*deletion *Le*YC36 mutant strain | This study |
| *Le*Δ*pilG* | *pilG-*deletion *Le*YC36 mutant strain | This study |
| *Le*Δ*pilS* | *pilS-*deletion *Le*YC36 mutant strain | This study |
| *Le*Δ*pilR* | *pilG-*deletion *Le*YC36 mutant strain | This study |
| *Le*Δ*pilW* | *pilW-*deletion *Le*YC36 mutant strain | This study |
| LssL-BL21 | *E. coli* BL21 (DE3) strain for recombinant LssL purification | This study |
| RltA-BL21 | *E. coli* BL21 (DE3) strain for recombinant RltA purification | This study |
| RltA_D52A_-BL21 | *E. coli* BL21 (DE3) strain for recombinant RltA_D52A_ purification | This study |
| RltB_psrr_-BL21 | *E. coli* BL21 (DE3) strain for recombinant RltB_psrr_ purification | This study |
| RltB_ir_-BL21 | *E. coli* BL21 (DE3) strain for recombinant RltB_ir_ purification | This study |
| **Plasmids** | **Relevant characteristics** | **Source** |
| pEX18Gm-*lssL* | The vector, carrying upstream and downstream sequences of *lssL*, used for *lssL* knock-out | This study |
| pEX18Gm-*Le*C3-*lssL* | The vector, carrying upstream and downstream sequences of *lssL*, used for *Le*C3-*lssL* knock-out | This study |
| pEX18Gm-*lssL-*com | The vector, carrying *lssL*, upstream and downstream sequences of *lssL*, used for *lssL* complementation | This study |
| pEX18Gm-*rltB* | The vector, carrying upstream and downstream sequences of *rltB*, used for *rltB* knock-out | This study |
| pEX18Gm-*rltA* | The vector, carrying upstream and downstream sequences of *rltA*, used for *rltA* knock-out | This study |
| pEX18Gm-*rltBA* | The vector, carrying upstream and downstream sequences of *rltBA*, used for *rltBA* knock-out | This study |
| pEX18Gm-*clpV-1* | The vector, carrying upstream and downstream sequences of *clpV-1*, used for *clpV-1* knock-out | This study |
| pEX18Gm-*hcp-1* | The vector, carrying upstream and downstream sequences of *hcp-1*, used for *hcp-1* knock-out | This study |
| pEX18Gm-*vgrG1-1* | The vector, carrying upstream and downstream sequences of *vgrG1-1*, used for *vgrG1-1* knock-out | This study |
| pEX18Gm-*pilG* | The vector, carrying upstream and downstream sequences of *pilG*, used for *pilG* knock-out | This study |
| pEX18Gm-*pilS* | The vector, carrying upstream and downstream sequences of *pilS*, used for *pilS* knock-out | This study |
| pEX18Gm-*pilR* | The vector, carrying upstream and downstream sequences of *pilR*, used for *pilR* knock-out | This study |
| pEX18Gm-*pilW* | The vector, carrying upstream and downstream sequences of *pilW*, used for *pilW* knock-out | This study |
| LssL-pET-28a(+) | The vector for recombinant LssL expression | This study |
| RltA-pET-28a(+) | The vector for recombinant RltA expression | This study |
| RltA_D52A_-pET-28a(+) | The vector for recombinant RltA_D52A_ expression | This study |
| RltB_psrr_-pET-28a(+) | The vector for recombinant RltB_psrr_ expression | This study |
| RltB_ir_-pET-28a(+) | The vector for recombinant RltB_ir_ expression | This study |

Table S2. All primers used in this study. These primers were used for gene in-frame deletion of *Le*YC36 or *Le*C3, recombinant protein purification, EMSA assays, and real-time PCR.

| **Primers** | **Sequence** | **Purpose** |
| --- | --- | --- |
| *lssL*-up-F-EcoR I | ccggaattcgccagcgacagcgcgtagcg | For gene in-frame deletion, completion, and in situ site-directed mutation |
| *lssL*-up-R | gagccaaccgtttcgacggacgggtggcgccg |  |
| *lssL*-down-F | gccacccgtccgtcgaaacggttggctcggacg |  |
| *lssL*-down-R-Hind Ⅲ | cccaagcttgcgtgttcgctgcgggtg |  |
| *rltB*-up-F-EcoR I | ccggaattccgatcctggaactgcaaccg |  |
| *rltB*-up-R | gctgcgcttactgcctcattcccacgcggcccg |  |
| *rltB*-down-F | cgcgtgggaatgaggcagtaagcgcagcgga |  |
| *rltB*-down-R-Hind Ⅲ | cccaagcttgagcacgaagctggtgctg |  |
| *rltA*-up-F-EcoR I | ccggaattcccgattcgctgaaggtctcgc |  |
| *rltA*-up-R | gcgctcattcccacgggatgtcggtcataaccac |  |
| *rltA*-down-F | ttatgaccgacatcccgtgggaatgagcgcgc |  |
| *rltA*-down-R-Hind Ⅲ | cccaagcttcaggtcgcgccagaaacggta |  |
| *rltBA*-up-R | cgctgcgcttactgcatgtcggtcataaccac |  |
| *rltBA*-down-F | ttatgaccgacatgcagtaagcgcagcgga |  |
| *clpV-1*-up-F-EcoR I | ccggaattcgtcgacggcgagggcgacgtgt |  |
| *clpV-1*-up-R | ttggccgcgccgatcgcggcaggtgtcgtcgagc |  |
| *clpV-1*-down-F | gacgacacctgccgcgatcggcgcggccaagag |  |
| *clpV-1*-down-R-Hind Ⅲ | cccaagcttcgcaggtcgtcgaccgcatcg |  |
| *hcp-1*-up-F-Hind Ⅲ | cccaagcttcttcatcccgctggtgcattg |  |
| *hcp-1*-up-R | cggtgtaggtgtccagtccttgtgatc |  |
| *hcp-1*-down-F | cacaaggactggacacctacaccgcgc |  |
| *hcp-1*-down-R-BamH I | cgcggatcctggccaggctcagggcggaga |  |
| *vgrG1-1*-up-F-Hind Ⅲ | cccaagcttccgtggatctgggtctggctt |  |
| *vgrG1-1*-up-R | acacctcggatgacccgatgccctggtctt |  |
| *vgrG1-1*-down-F | accagggcatcggggtcatccgaggtgtcac |  |
| *vgrG1-1*-down-R-BamH I | cgcggatcctctccgggcttctcggccccc |  |
| *Le*C3-*lssL*-up-F-EcoR I | gctatgaccatgattacgaattcgcctacctcgccaacga |  |
| *Le*C3-*lssL*-up-R | gccacccgtccgtcccacgggtgcggctcctgggt |  |
| *Le*C3-*lssL*-down-F | ggagccgcacccgtgggacggacgggtggcgccg |  |
| *Le*C3-*lssL*-down-R-Hind Ⅲ | acgacggccagtgccaagcttgccagcgacagcgcgtagc |  |
| *pilG*-up-F-EcoR I | gctatgaccatgattacgaattcgtactcggcgtcgaccg |  |
| *pilG*-up-R | cctaccccccggtcacattctttgtatccccca |  |
| *PilG*-down-F | gggatacaaagaatgtgaccggggggtagggca |  |
| *pilG*-down-R-Hind Ⅲ | acgacggccagtgccaagcttgtgagctgcggcagcgac |  |
| *pilS*-up-F-EcoR I | gctatgaccatgattacgaattcgccgactggtaggtctc |  |
| *pilS*-up-R | cacactgttcgctcacaagcggatttccctgcg |  |
| *PilS*-down-F | agggaaatccgcttgtgagcgaacagtgtgact |  |
| *pilS*-down-R-Hind Ⅲ | acgacggccagtgccaagcttgcgaccttgccgatggtc |  |
| *pilR*-up-F-EcoR I | gctatgaccatgattacgaattcgctcgcgcatgaacggc |  |
| *pilR*-up-R | aagcgccgctcagtcttcagccattacgccctcc |  |
| *PilR*-down-F | ggcgtaatggctgaagactgagcggcgcttgcg |  |
| *pilR*-down-R-Hind Ⅲ | acgacggccagtgccaagcttgccttgctgcttcttcat |  |
| *pilW*-up-F-EcoR I | gctatgaccatgattacgaattccggcgatcgcgtaatcg |  |
| *pilW*-up-R | ctccgacctatgaccaactgaccggagcacgcc |  |
| *PilW*-down-F | gtgctccggtcagttggtcataggtcggagctc |  |
| *pilW*-down-R-Hind Ⅲ | acgacggccagtgccaagcttgacgggttcccgcgccgc |  |
| *lssL*-F-BamH I | cgcggatccgcgccgaccttccagatgc | Recombinant protein purification and site-directed mutation |
| *lssL*-R-Hind Ⅲ | cccaagctttcaacagttcggcgccaccc |  |
| *rltB_psrr_*-F-BamH I | cgcggatcctgcatcaccgacgagaacagccg |  |
| *rltB_psrr_*-R-EcoR I | ccggaattccaggtccagttcgctccacggcac |  |
| *rltB_ir_*-F-BamH Ⅰ | cgcggatccatcgattcgatcgtgatcctgg |  |
| *rltB_ir_*-R-EcoR I | ccggaattcttactgcgccggcagatcga |  |
| *rltA*-F-EcoR I | ccggaattcatgaccgacatcctgctcatc |  |
| *rltA*-R-Hind Ⅲ | cccaagctttcattcccacgcggcccg |  |
| *rltA*_D52A_-F | gtggtgctgctggccgtgtcgctgcccg |  |
| *rltA*_D52A_-R | ctggccgggcagcgacacggccagcagc |  |
| P*_lssL_-*F | gcggcgtgcgcgtcgacgatgaaac | EMSA |
| P*_lssL_-*R | caaatgcagtacaagagcggcgcggga |  |
| P*_pilG_-*F | gacggatggtcttggaatcg |  |
| P*_pilG_-*R | gcggaatgcgtgcttgacag |  |
| P*_vgrG1-1_*-F | gtaatgccgcagccggtcgcaat |  |
| P*_vgrG1-1_*-R | gcattccttcgctgctcacgccgt |  |
| P*_hcp-1-clpV-1_*-F | gcacaggggccatccccaac |  |
| P*_hcp-1-clpV-1_*-R | gccatcggttcgcggaggtg |  |
| P*_Unrelated_*-F | gaatcgtcggccgccagggcg |  |
| P*_Unrelated_*-R | gcgagctcgaactcgtgatcgatg |  |
| q-16S-F | gtgcaagcgttactcggaat | For real-time PCR |
| q-16S-R | ctttcgtgcctcagtgtcag |  |
| q-*lssL*-F | gcgccgttgaacttgatctt |  |
| q-*lssL*-R | tacggcaagtacgtggtgat |  |
| q-*rltB*-F | cgaggcctacacctaccag |  |
| q-*rltB*-R | ccagaaacggtagaccagga |  |
| q-*rltA*-F | ctcatcgaagacgacgaacg |  |
| q-*rltA*-R | cagatctcgaacccgtcctg |  |
| q-*clpV-1*-F | gaataccaggaagcgcacag |  |
| q-*clpV-1*-R | ggtcaggatgatgagggtgt |  |
| q-*hcp-1*-F | ggcaaccaagaactgaagca |  |
| q-*hcp-1*-R | agatcggcttccttgacgat |  |
| q-*vgrG1-1*-F | cagcgaaggaatgcaacaga |  |
| q-*vgrG1-1*-R | caacacgtcgatccaccatt |  |
| q-*clpV-2*-F | cgtactcgacgtgggtttg |  |
| q-*clpV-2*-R | ggcttcgtcgatgaacaaga |  |
| q-*hcp-2*-F | gcaggacatctcgatcacca |  |
| q-*hcp-2*-R | gatggtcaggaagtcggtct |  |
| q-*vgrG-2*-F | actgccgcatcttcaagaac |  |
| q-*vgrG-2*-R | gcggtgtgggtgaagtagta |  |
| q-*pilG*-F | tctcagggtcatggtgatcg |  |
| q-*pilG*-R | aatgatttgcggctgttggt |  |
| q-*pilS*-F | tatctgttcatcgccagcct |  |
| q-*pilS*-R | ccgacgttgaacatcagcat |  |
| q-*pilR*-F | gccgtcctacatcgaagaga |  |
| q-*pilR*-R | atcccgagcttcttcagctt |  |

**Supplementary Movies**

**Movie S1. Movement and invasion of *L. enzymogenes* (*Le*) toward *S. aureus* (*Sa*), corresponding to Figure S1.** Real-time imaging was performed using a confocal laser microscope, with movie frames edited from images taken every 2 hours over a 48-hour period.

**Movies S2–S3. Movement behaviors of *Le* toward *Sa* at an initial distance of 4 μm.** Movie S2 shows a successful movement of *Le* toward *Sa* at the single-cell level with an initial distance of 4 μm, whereas Movie S3 shows a failed movement. The image in Fig. 1H is derived from Movie S2, and the image in Fig. 1J is from Movie S3. Movies S2 and S3 have a duration of 16 s and were recorded using a confocal microscope equipped with an optical tweezer system under a 60 × objective lens.

**References**

1.	Spottiswoode CN, Wood BM. Culturally determined interspecies communication between humans and honeyguides. *Science* 2023;**382**:1155–1158. https://doi.org/10.1126/science.adh4129

2.	Cosmo LG, Assis APA, de Aguiar MAM, *et al.* Indirect effects shape species fitness in coevolved mutualistic networks. *Nature* 2023;**619**:788–792. https://doi.org/10.1038/s41586-023-06319-7

3.	Wilde J, Slack E, Foster KR. Host control of the microbiome: mechanisms, evolution, and disease. *Science* 2024;**385**:eadi3338. https://doi.org/10.1126/science.adi3338

4.	Montoya JM, Pimm SL, Solé RV. Ecological networks and their fragility. *Nature* 2006;**442**:259–264. https://doi.org/10.1038/nature04927

5.	Cohen Y, Pasternak Z, Müller S, *et al.* Community and single cell analyses reveal complex predatory interactions between bacteria in high diversity systems. *Nat Commun* 2021;**12**:5481. https://doi.org/10.1038/s41467-021-25824-9

6.	Allesina S, Tang S. Stability criteria for complex ecosystems. *Nature* 2012;**483**:205–208. https://doi.org/10.1038/nature10832

7.	Lien Y-W, Amendola D, Lee KS, *et al.* Mechanism of bacterial predation via ixotrophy. *Science* 2024;**386**:eadp0614. https://doi.org/10.1126/science.adp0614

8.	Kaplan M, Chang Y-W, Oikonomou CM, *et al.* *Bdellovibrio* predation cycle characterized at nanometre-scale resolution with cryo-electron tomography. *Nat Microbiol* 2023;**8**:1267–1279. https://doi.org/10.1038/s41564-023-01401-2

9.	Zhang L, Guo L, Cui Z, *et al.* Exploiting predatory bacteria as biocontrol agents across ecosystems. *Trends Microbiol* 2024;**32**:398–409. https://doi.org/10.1016/j.tim.2023.10.005

10.	Fj C-M, A M-M, Fj M-T, *et al.* Siderophores and competition for iron govern myxobacterial predation dynamics. *ISME J* 2024;**18**:wrae077. https://doi.org/10.1093/ismejo/wrae077

11.	Rotem O, Pasternak Z, Shimoni E, *et al.* Cell-cycle progress in obligate predatory bacteria is dependent upon sequential sensing of prey recognition and prey quality cues. *Proc Natl Acad Sci U S A* 2015;**112**:E6028-6037. https://doi.org/10.1073/pnas.1515749112

12.	Sengupta A, Kruppa T, Löwen H. Chemotactic predator-prey dynamics. *Phys Rev E Stat Nonlin Soft Matter Phys* 2011;**83**:031914. https://doi.org/10.1103/PhysRevE.83.031914

13.	Lloyd DG, Whitworth DE. The Myxobacterium *Myxococcus xanthus* can sense and respond to the quorum signals secreted by potential prey organisms. *Front Microbiol* 2017;**8**:439. https://doi.org/10.3389/fmicb.2017.00439

14.	Westhoff S, van Wezel GP, Rozen DE. Distance-dependent danger responses in bacteria. *Curr Opin Microbiol* 2017;**36**:95–101. https://doi.org/10.1016/j.mib.2017.02.002

15.	Hungate BA, Marks JC, Power ME, *et al.* The functional significance of bacterial predators. *mBio* 2021;**12**:e00466-21. https://doi.org/10.1128/mBio.00466-21

16.	Wang Y, Qian G, Liu F, *et al.* Facile method for site-specific gene integration in *Lysobacter enzymogenes* for yield improvement of the anti-MRSA antibiotics WAP-8294A and the antifungal antibiotic HSAF. *ACS Synth Biol* 2013;**2**:670–678. https://doi.org/10.1021/sb4000806

17.	Zhang W, Li Y, Qian G, *et al.* Identification and characterization of the anti-methicillin-resistant *Staphylococcus aureus* WAP-8294A2 biosynthetic gene cluster from *Lysobacter enzymogenes* OH11. *Antimicrob Agents Chemother* 2011;**55**:5581–5589. https://doi.org/10.1128/AAC.05370-11

18.	Waso M, Khan S, Khan W. Assessment of predatory bacteria and prey interactions using culture-based methods and EMA-qPCR. *Microbiol Res* 2019;**228**:126305. https://doi.org/10.1016/j.micres.2019.126305

19.	Shen X, Wang B, Yang N, *et al.* *Lysobacter enzymogenes* antagonizes soilborne bacteria using the type IV secretion system. *Environ Microbiol* 2021;**23**:4673–4688. https://doi.org/10.1111/1462-2920.15662

20.	Yang M, Ren S, Shen D, *et al.* An intrinsic mechanism for coordinated production of the contact-dependent and contact-independent weapon systems in a soil bacterium. *PLoS Pathog* 2020;**16**:e1008967. https://doi.org/10.1371/journal.ppat.1008967

21.	Lou L, Qian G, Xie Y, *et al.* Biosynthesis of HSAF, a tetramic acid-containing macrolactam from *Lysobacter enzymogenes*. *J Am Chem Soc* 2011;**133**:643–645. https://doi.org/10.1021/ja105732c

22.	Xu H, Chen H, Shen Y, *et al.* Direct regulation of extracellular chitinase production by the transcription factor *Le*Clp in *Lysobacter enzymogenes* OH11. *Phytopathology* 2016;**106**:971–977. https://doi.org/10.1094/PHYTO-01-16-0001-R

23.	Fulano AM, Shen D, Kinoshita M, *et al.* The homologous components of flagellar type III protein apparatus have acquired a novel function to control twitching motility in a non-flagellated biocontrol bacterium. *Biomolecules* 2020;**10**:733. https://doi.org/10.3390/biom10050733

24.	Zhu Y, Han Y, Liu G, *et al.* Novel indole-mediated potassium ion import system confers a survival advantage to the *Xanthomonadaceae* family. *ISME J* 2022;**16**:1717–1729. https://doi.org/10.1038/s41396-022-01219-6

25.	Kang G, Allard CAH, Valencia-Montoya WA, *et al.* Sensory specializations drive octopus and squid behaviour. *Nature* 2023;**616**:378–383. https://doi.org/10.1038/s41586-023-05808-z

26.	Gomes DGE, Page RA, Geipel I, *et al.* Bats perceptually weight prey cues across sensory systems when hunting in noise. *Science* 2016;**353**:1277–1280. https://doi.org/10.1126/science.aaf7934

27.	Jakobsen L, Ratcliffe JM, Surlykke A. Convergent acoustic field of view in echolocating bats. *Nature* 2013;**493**:93–96. https://doi.org/10.1038/nature11664

28.	Clerc EE, Raina J-B, Keegstra JM, *et al.* Strong chemotaxis by marine bacteria towards polysaccharides is enhanced by the abundant organosulfur compound DMSP. *Nat Commun* 2023;**14**:8080. https://doi.org/10.1038/s41467-023-43143-z

29.	Raina J-B, Giardina M, Brumley DR, *et al.* Chemotaxis increases metabolic exchanges between marine picophytoplankton and heterotrophic bacteria. *Nat Microbiol* 2023;**8**:510–521. https://doi.org/10.1038/s41564-023-01327-9

30.	Wheeler JHR, Foster KR, Durham WM. Individual bacterial cells can use spatial sensing of chemical gradients to direct chemotaxis on surfaces. *Nat Microbiol* 2024;**9**:2308–2322. https://doi.org/10.1038/s41564-024-01729-3

31.	Keegstra JM, Carrara F, Stocker R. The ecological roles of bacterial chemotaxis. *Nat Rev Microbiol* 2022;**20**:491–504. https://doi.org/10.1038/s41579-022-00709-w

32.	Favre-Bulle IA, Scott EK. Optical tweezers across scales in cell biology. *Trends Cell Biol* 2022;**32**:932–946. https://doi.org/10.1016/j.tcb.2022.05.001

33.	Liu S, Qu Z, Zhao X, *et al.* Fast and versatile optical force measurement with digitally modulated stimulus in holographic optical tweezers. *Opt Laser Technol* 2023;**167**:109809. https://doi.org/10.1016/j.optlastec.2023.109809

34.	Tang B-L, Yang J, Chen X-L, *et al.* A predator-prey interaction between a marine *Pseudoalteromonas* sp. and Gram-positive bacteria. *Nat Commun* 2020;**11**:285. https://doi.org/10.1038/s41467-019-14133-x

35.	Wang M, Buist G, van Dijl JM. *Staphylococcus aureus* cell wall maintenance – the multifaceted roles of peptidoglycan hydrolases in bacterial growth, fitness, and virulence. *FEMS Microbiol Rev* 2022;**46**:fuac025. https://doi.org/10.1093/femsre/fuac025

36.	Lerner TR, Lovering AL, Bui NK, *et al.* Specialized peptidoglycan hydrolases sculpt the intra-bacterial niche of predatory *Bdellovibrio* and increase population fitness. *PLoS Pathog* 2012;**8**:e1002524. https://doi.org/10.1371/journal.ppat.1002524

37.	Yue H, Jiang J, Taylor AJ, *et al*. Outer membrane vesicle-mediated codelivery of the antifungal HSAF metabolites and lytic polysaccharide monooxygenase in the predatory *Lysobacter enzymogenes*. *ACS Chem Biol* 2021;**16**:1079–1089. https://doi.org/10.1021/acschembio.1c00260

38.	Banks EJ, Valdivia-Delgado M, Biboy J, *et al.* Asymmetric peptidoglycan editing generates cell curvature in *Bdellovibrio* predatory bacteria. *Nat Commun* 2022;**13**:1509. https://doi.org/10.1038/s41467-022-29007-y

39.	Razew A, Schwarz J-N, Mitkowski P, *et al.* One fold, many functions-M23 family of peptidoglycan hydrolases. *Front Microbiol* 2022;**13**:1036964. https://doi.org/10.3389/fmicb.2022.1036964

40.	Razew A, Laguri C, Vallet A, *et al.* *Staphylococcus aureus* sacculus mediates activities of M23 hydrolases. *Nat Commun* 2023;**14**:6706. https://doi.org/10.1038/s41467-023-42506-w

41.	Gonzalez-Delgado LS, Walters-Morgan H, Salamaga B, *et al.* Two-site recognition of *Staphylococcus aureus* peptidoglycan by lysostaphin SH3b. *Nat Chem Biol* 2020;**16**:24–30. https://doi.org/10.1038/s41589-019-0393-4

42.	Zhou X, Qian G, Chen Y, *et al.* PilG is involved in the regulation of twitching motility and antifungal antibiotic biosynthesis in the biological control agent *Lysobacter enzymogenes*. *Phytopathology* 2015;**105**:1318–1324. https://doi.org/10.1094/PHYTO-12-14-0361-R

43.	Xu K, Shen D, Yang N, *et al.* Coordinated control of the type IV pili and c-di-GMP-dependent antifungal antibiotic production in *Lysobacter* by the response regulator PilR. *Mol Plant Pathol* 2021;**22**:602–617. https://doi.org/10.1111/mpp.13046

44.	Jacob-Dubuisson F, Mechaly A, Betton J-M, *et al.* Structural insights into the signalling mechanisms of two-component systems. *Nat Rev Microbiol* 2018;**16**:585–593. https://doi.org/10.1038/s41579-018-0055-7

45.	Li D-Y, Han J-T, Zhang M-Y, *et al.* The two-component system RstA/RstB regulates expression of multiple efflux pumps and influences anaerobic nitrate respiration in *Pseudomonas fluorescens*. *mSystems* 2021;**6**:e0091121. https://doi.org/10.1128/mSystems.00911-21

46.	Qin X, Zhang K, Fan Y, *et al.* The bacterial MtrAB two-component system regulates the cell wall homeostasis responding to environmental alkaline stress. *Microbiol Spectr* 2022;**10**:e0231122. https://doi.org/10.1128/spectrum.02311-22

47.	Bayer-Santos E, Ceseti L de M, Farah CS, *et al.* Distribution, function and regulation of type 6 secretion systems of *Xanthomonadales*. *Front Microbiol* 2019;**10**:1635. https://doi.org/10.3389/fmicb.2019.01635

48.	MacIntyre DL, Miyata ST, Kitaoka M, *et al.* The *Vibrio cholerae* type VI secretion system displays antimicrobial properties. *Proc Natl Acad Sci U S A* 2010;**107**:19520–19524. https://doi.org/10.1073/pnas.1012931107

49.	Chou S, Bui NK, Russell AB, *et al.* Structure of a peptidoglycan amidase effector targeted to Gram-negative bacteria by the type VI secretion system. *Cell Rep* 2012;**1**:656–664. https://doi.org/10.1016/j.celrep.2012.05.016

50.	Le N-H, Pinedo V, Lopez J, *et al.* Killing of Gram-negative and Gram-positive bacteria by a bifunctional cell wall-targeting T6SS effector. *Proc Natl Acad Sci U S A* 2021;**118**:e2106555118. https://doi.org/10.1073/pnas.2106555118

51.	Schleifer KH, Kandler O. Peptidoglycan types of bacterial cell walls and their taxonomic implications. *Bacteriol Rev* 1972;**36**:407–477. https://doi.org/10.1128/br.36.4.407-477.1972

52.	Kudryakova I, Afoshin A, Tarlachkov S, *et al.* *Lysobacter gummosus* 10.1.1, a producer of antimicrobial agents. *Microorganisms* 2023;**11**:2853. https://doi.org/10.3390/microorganisms11122853

53.	Afoshin AS, Konstantinov MA, Toropygin IY, *et al*. β-lytic protease of *Lysobacter capsici* VKM B-2533T. *Antibiotics (Basel)* 2020;**9**:744. https://doi.org/10.3390/antibiotics9110744

54.	Li X, Pan C, Wang H, *et al.* Heterologous production of phenazines in the biocontrol agent *Lysobacter enzymogenes* C3. *J Agric Food Chem* 2025;**73**:1345–1355. https://doi.org/10.1021/acs.jafc.4c09518

55.	Ross AC, Xu Y, Lu L, *et al.* Biosynthetic multitasking facilitates thalassospiramide structural diversity in marine bacteria. *J Am Chem Soc* 2013;**135**:1155–1162. https://doi.org/10.1021/ja3119674

56.	Kayama G, Kanaly RA, Mori JF. Complete genome sequence of *Thalassospira* sp. strain GO-4, a marine bacterium isolated from a phenanthrene-enriched bacterial consortium. *Microbiol Resour Announc* 2022;**11**:e00532-22. https://doi.org/10.1128/mra.00532-22

57.	Lin L, Li L, Tao M, *et al.* Assembly of an active microbial consortium by engineering compatible combinations containing foreign and native biocontrol bacteria of kiwifruit. *Comput Struct Biotechnol J* 2023;**21**:3672–3679. https://doi.org/10.1016/j.csbj.2023.07.021

58.	Sabala I, Jagielska E, Bardelang PT, *et al.* Crystal structure of the antimicrobial peptidase lysostaphin from *Staphylococcus simulans*. *FEBS J* 2014;**281**:4112–4122. https://doi.org/10.1111/febs.12929

59.	Beaud Benyahia B, Taib N, Beloin C, *et al.* Terrabacteria: redefining bacterial envelope diversity, biogenesis and evolution. *Nat Rev Microbiol* 2025;**23**:41-56. https://doi.org/10.1038/s41579-024-01088-0

60.	Zhang S, Schlabach K, Pérez Carrillo VH, *et al.* A chemical radar allows bacteria to detect and kill predators. *Cell* 2025;**188**:2495-2504.e20. https://doi.org/10.1016/j.cell.2025.02.033

61.	Alyuruk H, Kontas A. Dissolved free, total and particulate enantiomeric amino acid levels in eutrophic and oligotrophic parts of a semi-enclosed bay (İzmir, Aegean Sea). *Reg Stud Mar Sci* 2021;**44**:101750. https://doi.org/10.1016/j.rsma.2021.101750

62.	Huang J, Yu S. Analysis of 20 free amino acids in a Shanghai drinking water treatment plant using solid-phase extraction coupled with liquid chromatography-tandem mass spectrometry. *Acad J Sci Technol* 2025;**15**:267–272. https://doi.org/10.54097/d7k31591

63.	Trevisan F, Waschgler F, Tiziani R, *et al.* Exploring glycine root uptake dynamics in phosphorus and iron deficient tomato plants during the initial stages of plant development. *BMC Plant Biol* 2024;**24**:495. https://doi.org/10.1186/s12870-024-05120-6

64.	Kuzyakov Y, Blagodatskaya E. Microbial hotspots and hot moments in soil: concept & review. *Soil Biology and Biochemistry* 2015;**83**:184–199. https://doi.org/10.1016/j.soilbio.2015.01.025

65.	Wang J, Hashem I, Bhonsale S, *et al.* Individual-based modeling (IbM) unravels spatial and social interactions in bacterial communities. *ISME J* 2025;wraf116. https://doi.org/10.1093/ismejo/wraf116

66.	Booth SC, Meacock OJ, Foster KR. Cell motility empowers bacterial contact weapons. *ISME J* 2024;**18**:wrae141. https://doi.org/10.1093/ismejo/wrae141

67.	Lin L, Xu K, Shen D, *et al.* Antifungal weapons of *Lysobacter*, a mighty biocontrol agent. *Environ Microbiol* 2021;**23**:5704–5715. https://doi.org/10.1111/1462-2920.15674

68.	Chen Y, Yu L, Liu F, *et al.* Spermidine-regulated biosynthesis of Heat-Stable Antifungal Factor (HSAF) in *Lysobacter enzymogenes* OH11. *Front Microbiol* 2018;**9**:2984. https://doi.org/10.3389/fmicb.2018.02984

69.	Chandra Ojha S, Imtong C, Meetum K, *et al.* Purification and characterization of the antibacterial peptidase lysostaphin from *Staphylococcus simulans*: adverse influence of Zn^2+^ on bacteriolytic activity. *Protein Expres Purif* 2018;**151**:106–112. https://doi.org/10.1016/j.pep.2018.06.013

70.	Han Y, Wang Y, Yu Y, *et al.* Indole-induced reversion of intrinsic multiantibiotic resistance in *Lysobacter enzymogenes*. *Appl Environ Microbiol* 2017;**83**:e00995-17. https://doi.org/10.1128/AEM.00995-17

71.	Pasquina-Lemonche L, Burns J, Turner RD, *et al.* The architecture of the Gram positive bacterial cell wall. *Nature* 2020;**582**:294–297. https://doi.org/10.1038/s41586-020-2236-6

72.	Turner RD, Mesnage S, Hobbs JK, *et al.* Molecular imaging of glycan chains couples cell-wall polysaccharide architecture to bacterial cell morphology. *Nature Communications* 2018;**9**. https://doi.org/10.1038/s41467-018-03551-y

73.	Turner RD, Hobbs JK, Foster SJ. Atomic force microscopy analysis of bacterial cell wall peptidoglycan architecture. *Methods Mol Biol* 2016;**1440**:3–9. https://doi.org/10.1007/978-1-4939-3676-2_1

74.	Małecki PH, Mitkowski P, Jagielska E, *et al.* Structural characterization of EnpA D, L-endopeptidase from *Enterococcus faecalis* prophage provides insights into substrate specificity of M23 peptidases. *Int J Mol Sci* 2021;**22**:7136. https://doi.org/10.3390/ijms22137136

75.	Lin L, Zhou M, Shen D, *et al.* A non-flagellated biocontrol bacterium employs a PilZ-PilB complex to provoke twitching motility associated with its predation behavior. *Phytopathology Research* 2020;**2**:12. https://doi.org/10.1186/s42483-020-00054-x

76.	Pérez J, Jiménez-Zurdo JI, Martínez-Abarca F, *et al.* Rhizobial galactoglucan determines the predatory pattern of *Myxococcus xanthus* and protects *Sinorhizobium meliloti* from predation. *Environ Microbiol* 2014;**16**:2341–2350. https://doi.org/10.1111/1462-2920.12477

77.	Wang D, Zhu L, Zhen X, *et al.* A secreted effector with a dual role as a toxin and as a transcriptional factor. *Nat Commun* 2022;**13**:7779. https://doi.org/10.1038/s41467-022-35522-9

78.	Qu Z, Wang K, Feng C, *et al.* Optofluidic sorting of microparticles using Airy beams. *Int J Optomechatroni* 2024;**18**:2375538. https://doi.org/10.1080/15599612.2024.2375538

79.	Ge A, Wang X, Ge M, *et al.* Profile analysis of *C. elegans* rheotaxis behavior using a microfluidic device. *Lab Chip* 2019;**19**:475–483. https://doi.org/10.1039/c8lc01087k

80.	Henshaw RJ, Moon J, Stehnach MR, *et al.* Metabolites from intact phage-infected *Synechococcus* chemotactically attract heterotrophic marine bacteria. *Nat Microbiol* 2024;**9**:3184–3195. https://doi.org/10.1038/s41564-024-01843-2

81.	Hu P, Ly KL, Pham LPH, *et al.* Bacterial chemotaxis in static gradients quantified in a biopolymer membrane-integrated microfluidic platform. *Lab Chip* 2022;**22**:3203–3216. https://doi.org/10.1039/d2lc00481j

82.	Bothe S, Hänzelmann P, Böhler S, *et al.* Fragment screening using biolayer interferometry reveals ligands targeting the SHP-motif binding site of the AAA+ ATPase p97. *Commun Chem* 2022;**5**:169. https://doi.org/10.1038/s42004-022-00782-5

83.	Chen J, Zhou Y, Liu Z, *et al.* Hepatic glycogenesis antagonizes lipogenesis by blocking S1P via UDPG. *Science* 2024;**383**:eadi3332. https://doi.org/10.1126/science.adi3332

84.	Fan R, Li Z, Shi X, *et al.* Expression, purification, and characterization of the recombinant, two-component, response regulator ArlR from *Fusobacterium nucleatum.* *Appl Biochem Biotechnol* 2022;**194**:2093–2107. https://doi.org/10.1007/s12010-021-03785-5

85.	Liu B, Liu Y, Yang B, *et al. Escherichia coli* O157:H7 senses microbiota-produced riboflavin to increase its virulence in the gut. *Proc Natl Acad Sci U S A* 2022;**119**:e2212436119. https://doi.org/10.1073/pnas.2212436119

86.	Lin H, Deng E-Z, Ding H, *et al*. iPro54-PseKNC: a sequence-based predictor for identifying sigma-54 promoters in prokaryote with pseudo k-tuple nucleotide composition. *Nucleic Acids Res* 2014;**42**:12961–12972. https://doi.org/10.1093/nar/gku1019

87.	Reese MG. Application of a time-delay neural network to promoter annotation in the *Drosophila melanogaster* genome. *Comput Chem* 2001;**26**:51–56. https://doi.org/10.1016/s0097-8485(01)00099-7

88.	Letunic I, Khedkar S, Bork P. SMART: recent updates, new developments and status in 2020. *Nucleic Acids Res* 2021;**49**:D458–D460. https://doi.org/10.1093/nar/gkaa937

89.	Hallgren J, Tsirigos KD, Pedersen MD, *et al.* DeepTMHMM predicts alpha and beta transmembrane proteins using deep neural networks. *bioRxiv* 2022. https://doi.org/10.1101/2022.04.08.487609

90.	Teufel F, Almagro Armenteros JJ, Johansen AR, *et al.* SignalP 6.0 predicts all five types of signal peptides using protein language models. *Nat Biotechnol* 2022;**40**:1023–1025. https://doi.org/10.1038/s41587-021-01156-3

91.	Schoch CL, Ciufo S, Domrachev M, *et al.* NCBI Taxonomy: a comprehensive update on curation, resources and tools. *Database* 2020;**2020**:baaa062. https://doi.org/10.1093/database/baaa062

92.	Chen I-MA, Chu K, Palaniappan K, *et al.* The IMG/M data management and analysis system v.7: content updates and new features. *Nucleic Acids Res* 2023;**51**:D723–D732. https://doi.org/10.1093/nar/gkac976

93.	Rozewicki J, Li S, Amada KM, *et al.* MAFFT-DASH: integrated protein sequence and structural alignment. *Nucleic Acids Res* 2019;**47**:W5–W10. https://doi.org/10.1093/nar/gkz342

94.	Capella-Gutiérrez S, Silla-Martínez JM, Gabaldón T. trimAl: a tool for automated alignment trimming in large-scale phylogenetic analyses. *Bioinformatics* 2009;**25**:1972–1973. https://doi.org/10.1093/bioinformatics/btp348

95.	Minh BQ, Schmidt HA, Chernomor O, *et al.* IQ-TREE 2: new models and efficient methods for phylogenetic inference in the genomic era. *Mol Biol Evol* 2020;**37**:1530–1534. https://doi.org/10.1093/molbev/msaa015

96.	Letunic I, Bork P. Interactive tree of life (iTOL) v3: an online tool for the display and annotation of phylogenetic and other trees. *Nucleic Acids Res* 2016;**44**:W242–W245. https://doi.org/10.1093/nar/gkw290
